# A multi-strategy enhanced secretary bird optimization algorithm for high-precision inverse kinematics in robotic arms

**DOI:** 10.1371/journal.pone.0331041

**Published:** 2025-08-29

**Authors:** Xuhao Chen, Nuohan Lin, Fan Zhang, Xuhai Zhao

**Affiliations:** 1 Southwest University, College of Engineering and Technology, Chongqing, China; 2 Tongji University, The School of Aerospace Engineering and Applied Mechanics, Shanghai, China; 3 Chongqing University of Posts and Telecommunications, International college, Chongqing, China; 4 Guangdong Baiyun University, School of Accounting, Guangzhou, China; Southeast University, CHINA

## Abstract

The assembly of pyrotechnic grain demands high precision and stability in robotic arm motion control due to the small shell apertures and stringent assembly accuracy requirements. Inverse kinematics is a core technology in robotic arm motion control. This paper constructs a robotic arm inverse kinematics model by reformulating the inverse kinematics problem as a constrained optimization problem and employs a multi-strategy improved Secretary Bird Optimization Algorithm (ISBOA) to achieve high-precision solutions. Aiming at the problems of restricted solution set exploration, easy to fall into local optimization and slow convergence when solving the inverse kinematics of multi-DOF robotic arm by SBOA, this paper introduces the oppositional variational perturbation, golden sine development and evolutionary strategy to optimize the formation of ISBOA, and verifies its effectiveness through numerical experiments. Simulation experiments using 4, 6, and 7-DOF robotic arms are conducted, with inverse solution results analyzed via PCA dimensionality reduction and K-means clustering, demonstrating the superiority of ISBOA in inverse solution diversity. Finally, a MATLAB-CoppeliaSim-UR16e experimental platform is developed to compare ISBOA with traditional analytical and Newton iterative method. Results are analyzed in terms of assembly accuracy, singular position handling, grasping success rate, and assembly success rate, confirming ISBOA’s advantages in pyrotechnic grain assembly and its potential for engineering applications.

## 1. Introduction

Pyrotechnics is a specialized and highly complex field within the manufacturing industry, characterized by significant risks [1]. The production and assembly processes of pyrotechnic products demand advanced technological capabilities. With the continuous advancement of manufacturing technologies, the variety and demand for pyrotechnic products have been steadily increasing [2]. However, the traditional assembly method overly relies on manual operation, which has problems such as low efficiency, insufficient safety and lack of flexibility. Especially when handling high-energy substances, the accidental action of mechanical, thermal or electrical energy may trigger an explosion, which seriously threatens the life safety of the operators while increasing the uncertainty and danger of production [3,4]. As a result, integrating intelligent technologies to enhance the precision, flexibility, and safety of pyrotechnic assembly processes has emerged as a critical challenge.

**Fig 1:**
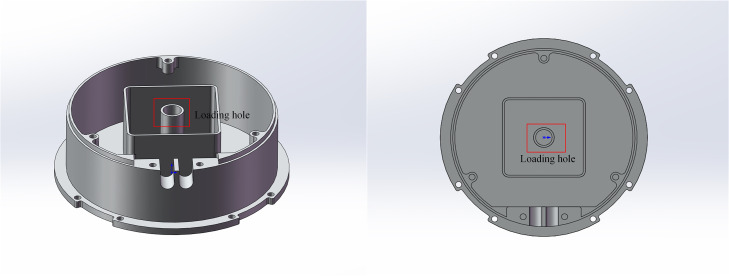
Pyrotechnic shells.

This study focuses on the assembly of pyrotechnic grain at an enterprise (see Fig 1). These products are characterized by narrow shell apertures, stringent assembly precision requirements, which impose exceptionally high demands on the accuracy and stability of robotic arm motion control. Among them, inverse kinematics solving is the core technology of robotic arm motion control [5–7], which can accurately map the target position to the joint angle, and its accuracy and real-time directly affect the operating efficiency of the robotic arm and product quality. Traditional inverse kinematics solutions primarily rely on analytical methods [8,9], which simplify multi-dimensional matrix computations. However, these methods are heavily dependent on the geometric parameters of the robotic arm and require compliance with the Pieper criterion [10]. The complex one-to-many mapping relationship between the robotic arm’s position and joint angles further complicates the calculation of inverse solutions. Additionally, traditional approaches are prone to singularity issues, where singular configurations may lead to computational failures or unstable results, thereby restricting the application of inverse kinematics in flexible manufacturing [11]. Consequently, developing an efficient and robust inverse kinematics solution method that can adapt to diverse industrial scenarios is essential for addressing the complexities of modern manufacturing demands.

Traditional inverse kinematics solution methods for robotic arms are mainly classified into analytical [12,13] and numerical methods [14], each of which has its own characteristics. The analytical method, also referred to as the closed-form solution method, directly addresses the inverse kinematics problem by leveraging geometric relationships or algebraic transformations. The method derives equations based on the structure of the robot arm, and multiple solutions can be derived without initial values, and then a unique solution is selected based on specific conditions. The primary advantage of the analytical method lies in its computational speed and efficiency, making it particularly suitable for robotic arm operations that demand real-time control. However, it imposes strict requirements on the robotic arm’s structure and is only applicable to configurations with specific geometric characteristics, such as those meeting the Pieper criterion. These limitations reduce its applicability in more complex scenarios. Zhao Guojun et al. [15]proposed a closed-form analytical method that identifies redundant joints by comparing the workspace volumes of various fixed joints and derives closed-form expressions for nonparametric joint angles. To address singularity and coupling effects in 3-sphere parallel manipulators (RSPM), Ahmadi N. Ali [16] developed an algorithm based on the closed-loop vector method. Similarly, for SSRMS-type space manipulators with 7 degrees of freedom, Jingdong Zhao [17] introduced an analytical inverse kinematics approach called triple-continuous parallel link directionally-adapted parameterization (LDVP) within the framework of conformal geometry. This method uses the axial direction vectors of parallel joints 3, 4, and 5 for parameterization and has been experimentally verified to achieve an average solution time of 9.79 microseconds in the forward direction and 0.25 milliseconds in the inverse direction. Despite its advantages, the analytical method has notable limitations. It is constrained to robotic arms with specific configurations and geometries, lacks flexibility, and cannot effectively address prioritized constraints such as obstacle avoidance. Furthermore, the solution process can be complex and error-prone, particularly for arms that do not satisfy the Pieper criterion. For robotic arms with redundant structures or multiple degrees of freedom, the analytical method becomes less practical [18–20]. In such cases, numerical solution methods are recommended for solving inverse kinematics problems.

Numerical solution methods transform the inverse kinematics problem of a redundant robotic arm into an optimization task, where the desired solution is obtained through iterative methods and objective functions. Common numerical approaches include Newton iterative method [21], the Jacobian matrix method [7], and various optimization techniques. These methods are less dependent on the robotic arm’s configuration and geometric parameters, offering flexibility and generalizability in their computational processes. Yang Xiaofeng [22] proposed a novel inverse kinematics solution that combines the strengths of the Jacobian matrix method and the analytical method to address the inverse kinematics of robotic arms with biased joints. Colan Jacinto et al. [23] studied the impact of step size selection on the Jacobian matrix-based inverse kinematics of robotic arms. Their experiments demonstrated that variable step-size strategies can enhance solution performance and show potential for broader applications. Despite their advantages, numerical methods involve a trade-off between solution speed and accuracy, often making it difficult to simultaneously meet real-time and high-precision requirements [24]. Moreover, the choice of initial values significantly affects convergence, resulting in slower solution speeds compared to analytical methods. In recent years, advancements in computer technology and algorithms have enabled researchers to integrate optimization algorithms into the inverse kinematics problem of robotic arms [25–28]. These developments have significantly improved both computational efficiency and accuracy, providing new avenues for the advancement of robotic systems.

Optimization methods for solving inverse kinematics problems are generally classified into two main categories: meta-heuristic algorithms and neural networks. Meta-heuristic algorithms have been extensively applied to improve the performance of redundant robotic arms. Ma Wenli [29] proposed a modified quantum particle swarm optimization algorithm (DMII-IQPSO) based on isotropic exponential constraints for directional maneuvering, addressing solution errors and singular configurations in the inverse kinematics of a seven-degree-of-freedom (7-DOF) redundant robotic arm. Kaur Manpreet [30] utilized a Coyote optimization algorithm for optimizing the inverse kinematics of a 7-DOF robotic arm. To address the redundant inverse kinematics problem in 5-DOF robots, Huang X.C. [31] introduced a bio-inspired DNA algorithm, which simulation results demonstrated to outperform traditional genetic and particle swarm algorithms. Hernandez-Barragan Jesus [32] proposed a multimodal firefly algorithm for solving inverse solutions, enabling a broader range of joint configurations to achieve the same end-effector pose. This approach improved the performance of classical algorithms and was validated through real-time experiments on a 5-DOF robot. Seyyedabbasi Amir [33] applied a sand cat swarm optimization algorithm to the inverse kinematics of a 6-DOF PUMA robotic arm, comparing its performance with the particle swarm, grey wolf, and whale optimization algorithms. Masthan S.A.R. Sheik [27] introduced a hybrid optimization algorithm, e^3^GSA, based on the gravity search algorithm, to solve the inverse kinematics of multi-link redundant robotic arms. Simulation experiments showed that this algorithm achieved faster convergence and shorter computation times in robotic arms with 6, 8, and 12-DOF. Rokbani Nizar [34] proposed an SSA algorithm based on β distribution, with inverse motion experiments on an 8-DOF robotic arm demonstrating superior performance compared to other algorithms. Meta-heuristic algorithms exhibit high efficiency and accuracy in solving multivariate multimodal functions, significantly enhancing the performance of redundant robotic arms. However, their lack of real-time performance limits their applicability in scenarios that demand fast response times. The emergence of neural networks has introduced new possibilities for solving inverse kinematics problems, offering potential improvements in real-time performance and adaptability.

Omur Aydogmus et al. [35] developed a deep neural network optimized using hyper-parametric Bayesian optimization, generating 10 billion data samples in a simulation environment. After training, the network was tested on simulated and real six-degree-of-freedom humanoid robots, achieving excellent control predictions within 25 milliseconds. Rania Bouzid et al. [36] constructed an artificial neural network to solve the inverse kinematics problem of a four-degree-of-freedom SCARA robot, aiming to minimize the mean square error (MSE). By comparing different training methods and datasets, they identified the optimal neural network training configuration, significantly improving computational efficiency and accuracy. Hsieh Yi-Zeng et al. [26] proposed a Deep Convolutional Generative Adversarial Network (DCGAKN) to model the inverse kinematics of self-assembling robotic arms. This approach addresses the challenges of limited solution space and enhances adaptability in dynamic environments. Similarly, Cagigas-Muniz Daniel [25] applied an artificial neural network to solve the inverse kinematics problem of articulated robots. His proposed bootstrap sampling and hybrid methods improved network performance. By learning the data distribution, the neural network achieves real-time control of the robotic arm without relying on traditional inverse kinematics equations. This approach greatly simplifies the solution process for complex problems, offering a new avenue for the flexibility and intelligent development of robotic systems. However, neural networks come with high training costs and are significantly influenced by the quality and quantity of datasets.

Table 1 summarizes the advantages and disadvantages of the aforementioned inverse kinematics solution methods for robotic arms. For the specialized scenario of pyrotechnic grain assembly, considering the small aperture diameter of the pyrotechnic product shells and the high assembly accuracy requirements, the inverse kinematics model for the assembly robotic arm is established and solved using an optimization algorithm. After evaluating solving accuracy, convergence speed, and computational cost, this approach is deemed more practical and applicable for the task.

**Table 1:** Summary of inverse kinematics solution methods.

Method Categories			Advantages	Disadvantages
Analytical method			Fast computation speed for real-time control	The robotic arm configuration must satisfy the Pieper criterion, with multiple or no solutions present
Numerical method	Newton iterative method		Fast convergence	Dependent on the initial solution case, or converge to a local optimum
	Jacobi matrix method		Suitable for multi-DOF robotic arms, capable of handling redundancy problems	Possible singularities in the Jacobi matrix, leading to instability of the algorithm
	Optimization method	Metaheuristic algorithms	Handles highly nonlinear, unanalyzed problems with high inverse solution accuracy	High computational volume, poor real-time performance
		Neural network	Handles highly nonlinear problems without analytic solutions	Large training cost, susceptible to the quantity and quality of the dataset

In summary, the establishment of a mathematical model for the inverse kinematics problem of a robotic arm effectively transforms the problem into an optimal control problem. The solution obtained under this framework is the inverse solution of the robotic arm when all constraints are satisfied. Building on this, this paper conducts an in-depth analysis of an optimization algorithm named Secretary Bird Optimization Algorithm (SBOA) [37], proposed in 2024. The results reveal that SBOA suffers from several limitations, including restricted exploration of the solution space, slow convergence, and a tendency to fall into local optima when solving the inverse kinematics problem of multi-DOF robotic arms. These shortcomings necessitate further research and improvement. To address these challenges, this paper introduces a multi-strategy enhanced ISBOA algorithm tailored specifically for solving robotic inverse kinematics problems. The main contributions of this paper are summarized as follows:

(1) To overcome the limitations of the SBOA algorithm in solution space exploration, convergence speed, and susceptibility to local optima, this paper incorporates three strategies: the oppositional variational perturbation strategy, the golden sine development strategy, and the evolutionary strategy. These enhancements result in the development of a new ISBOA algorithm.
(2) Numerical experiments using the CEC2017 test set demonstrate that the ISBOA algorithm achieves faster convergence and higher computational accuracy compared to the original SBOA algorithm. Furthermore, when compared with seven other prominent optimization algorithms from recent years, ISBOA exhibits strong competitiveness.
(3) An inverse kinematics solution model based on the ISBOA algorithm is constructed and validated through simulation experiments using robotic arms with 4, 6, and 7-DOF. The ISBOA algorithm outperforms other optimization algorithms in terms of convergence curves and computational accuracy. Additionally, principal component analysis (PCA) is applied to reduce the dimensionality of the inverse solution results, which are then analyzed using the K-means clustering method. This demonstrates the diversity of the inverse solution results achieved by the ISBOA algorithm, while maintaining a high degree of accuracy.
(4) A MATLAB-CoppeliaSim-UR16e experimental platform for pyrotechnic grain assembly is developed. Simulation experiments are conducted in MATLAB-CoppeliaSim, followed by physical validation using a UR16e robotic arm. The ISBOA algorithm is compared with traditional analytical methods in terms of assembly accuracy, singular positional attitudes, gripping success rate, and assembly success rate. The results indicate that the inverse kinematics solution model based on the ISBOA algorithm exhibits superior performance and feasibility in the assembly process of pyrotechnic grain, demonstrating its engineering significance.

**Fig 2:**
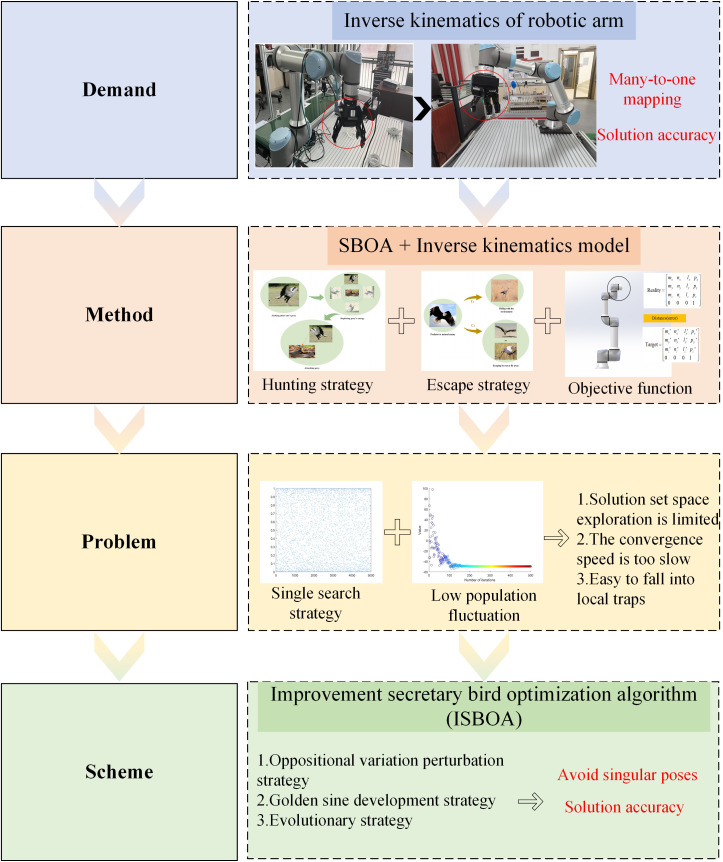
Motivation for the paper.

Fig 2 visualizes the motivation of this study, illustrating the research concept and its value. The structure of this paper is organized as follows: Section 2 introduces the related theoretical work, including the derivation of the kinematic equations of the assembly robotic arm, the establishment of the inverse kinematics objective function, and an elaboration on the original SBOA algorithm. Section 3 presents the ISBOA algorithm and its three strategic improvements. Section 4 details the experimental results and analysis, including numerical experiments using the CEC2017 test set, simulation experiments on the inverse kinematics of multi-DOF robotic arms, and physical validation for the assembly of pyrotechnic grain. Section 5 provides the summary and outlook.

## 2. Related theoretical work

### 2.1 Kinematic analysis of the assembly robotic arm

**Fig 3:**
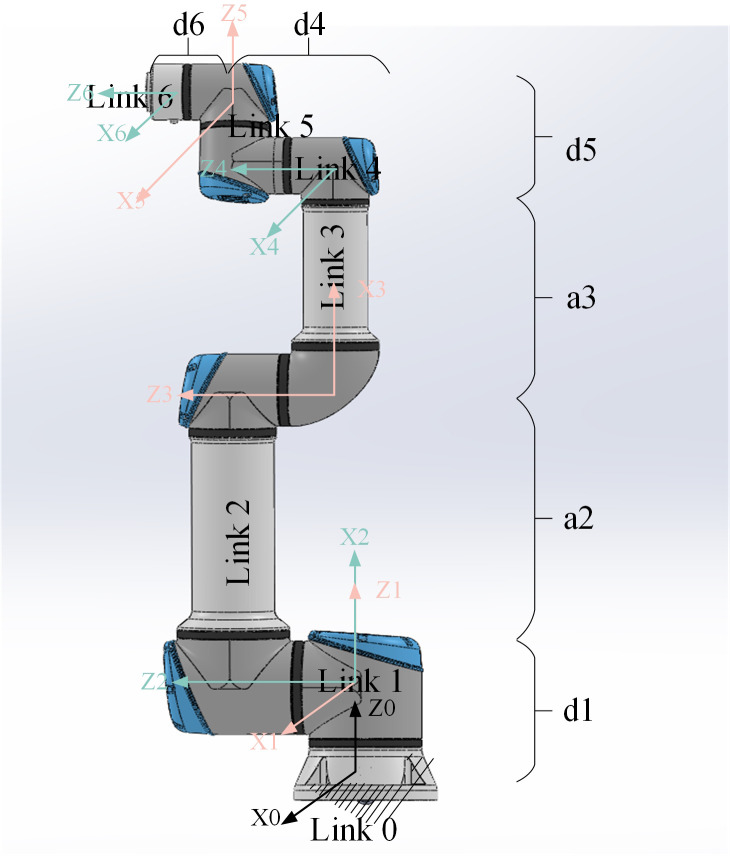
DH modeling of an assembly robotic arm.

The mapping from the joint space of a robotic arm to its workspace is referred to as forward kinematics. This process is used to determine the position and orientation of the end-effector within the workspace. Conversely, the mapping that derives the joint parameters from the workspace is known as inverse kinematics, which is primarily utilized for planning the trajectory of the robotic arm. Together, these two mappings form the theoretical foundation of robotic arm control. Forward kinematics is also referred to as kinematic modeling, while inverse kinematics is termed kinematic solving. The core of kinematic modeling lies in constructing an accurate mathematical model that captures the geometric relationships between the robotic arm’s joints, as well as the position and orientation of the end-effector within a global coordinate system. Common methods for kinematic modeling include geometric modeling and the Denavit-Hartenberg (DH) modeling technique. Owing to its high versatility, the DH modeling method has become a standard approach for the kinematic analysis of robotic arms. It is suitable for most series and parallel robotic arm structures, and it represents the arm using standardized parameters: joint angle, link length, offset, and torsion angle. This method simplifies the complex structure of a robotic arm into a clear and concise mathematical framework. The DH modeling method not only facilitates the establishment and solution of kinematic equations but can also be directly applied to the design and implementation of control systems. Fig 3 illustrates the DH modeling of the UR16e assembly robotic arm.

$A_{i-1}^{i}$ between adjacent joints of the robotic arm.


$$\begin{array}{c} A_{i-1}^{i}=Trans(0,0,a_{i})Rot(Z,b_{i})Trans(c_{i},0,0)Rot(X,d_{i})\\ =\left[\begin{array}{c} \cos a_{i}\;\;\;\;\;-\sin a_{i}\cos d_{i}\;\;\;\;\;\;\;\sin a_{i}\sin d_{i}\;\;\;\;\;\;\;\;\;\;\;c_{i}\cos a_{i}\\ \sin a_{i}\;\;\;\;\;\;\cos a_{i}\cos d_{i}\;\;\;\;\;\;\;\;\;-\cos a_{i}\sin d_{i}\;\;\;\;\;\;c_{i}\sin a_{i}\\ \;\;\;0\;\;\;\;\;\;\;\;\;\;\;\;\;\;\;\;\;\;\sin d_{i}\;\;\;\;\;\;\;\;\;\;\;\;\;\;\;\;\;\;\;\cos d_{i}\;\;\;\;\;\;\;\;\;\;\;\;\;\;\;\;\;\;\;b_{i}\\ \;\;\;0\;\;\;\;\;\;\;\;\;\;\;\;\;\;\;\;\;\;\;\;\;0\;\;\;\;\;\;\;\;\;\;\;\;\;\;\;\;\;\;\;\;\;\;\;\;\;\;\;\;0\;\;\;\;\;\;\;\;\;\;\;\;\;\;\;\;\;\;\;\;\;\;\;\;\;1 \end{array}\right] \end{array}$$ (1)

$$A_{0}^{6}=A_{0}^{1}A_{1}^{2}A_{2}^{3}A_{3}^{4}A_{4}^{5}A_{5}^{6}=\left[\begin{array}{c} f_{x}\;\;\;\;\;g_{x}\;\;\;\;\;h_{x}\;\;\;\;j_{x}\\ f_{y}\;\;\;\;\;g_{y}\;\;\;\;h_{y}\;\;\;\;j_{y}\\ f_{z}\;\;\;\;\;g_{z}\;\;\;\;\;h_{z}\;\;\;\;j_{z}\\ 0\;\;\;\;\;\;\;\;0\;\;\;\;\;\;\;0\;\;\;\;\;\;\;1 \end{array}\right]=\left[\begin{array}{c} R_{3\times 3}\;\;\;\;P_{1\times 3}\\ 0_{1\times 3}\;\;\;\;\;\;\;1 \end{array}\right]$$ (2)

In the DH modeling method, *a* represents the joint angle, *b* represents link length, *c* represents the offset, and *d* represents the torsion angle. Using these parameters, the forward kinematics of the robotic arm can be deduced as shown in Equation 1 and Equation 2. During the gripping and transporting processes of the robotic arm, its actions can be described as rotations *[R*_*X*_*, R*_*Y*_*, R*_*Z*_*]* about the X, Y, and Z axes, and translations of the target point *[P*_*X*_*, P*_*Y*_*, P*_*Z*_*]*. By combining the joint motion angles with the transformation matrix, the coordinate transformation between the base of the robotic arm and the end-effector can be achieved.

### 2.2 Inverse kinematics objective function

This paper focuses on the development of flexible automatic assembly technology for multi-species and small-batch pyrotechnic products, addressing key challenges such as low automation levels and difficulties in ensuring safety during the current pyrotechnic assembly processes. Due to the small aperture of the outer shell and the need for high assembly precision, the robotic arm must accurately reach the target point during the assembly process, placing stringent demands on the kinematic solution. However, traditional inverse kinematics methods primarily rely on simplified multi-dimensional matrix computations, which suffer from high computational complexity and are unsuitable for highly redundant and non-standard robotic arms. These limitations significantly hinder the advancement of automated pyrotechnic assembly. To overcome these challenges, this paper reformulates the inverse kinematics problem as a constrained optimization problem and solves it using an optimization-based approach. This method achieves efficient, fast, and high-precision inverse kinematics computation, offering a novel framework for the design and application of future robotic systems.

The kinematic model of the assembly robotic arm is established in Section 2.1, where the target position of the robotic arm, *A*_*tar*_, is defined in relation to the actual position, *A*_*act*_, as shown in Equation 3.


$$A_{act}=\left[\begin{array}{c} f_{x}^{e}\;\;\;\;g_{x}^{e}\;\;\;\;\;h_{x}^{e}\;\;\;\;j_{x}^{e}\\ f_{y}^{e}\;\;\;\;g_{y}^{e}\;\;\;\;h_{y}^{e}\;\;\;\;j_{y}^{e}\\ f_{z}^{e}\;\;\;\;g_{z}^{e}\;\;\;\;h_{z}^{e}\;\;\;\;j_{z}^{e}\\ 0\;\;\;\;\;\;\;0\;\;\;\;\;\;\;0\;\;\;\;\;\;\;1 \end{array}\right],A_{tar}=\left[\begin{array}{c} f_{x}\;\;\;\;\;g_{x}\;\;\;\;\;h_{x}\;\;\;\;j_{x}\\ f_{y}\;\;\;\;\;g_{y}\;\;\;\;h_{y}\;\;\;\;j_{y}\\ f_{z}\;\;\;\;\;g_{z}\;\;\;\;h_{z}\;\;\;\;j_{z}\\ 0\;\;\;\;\;\;\;\;0\;\;\;\;\;\;0\;\;\;\;\;\;\;1 \end{array}\right]$$ (3)

To minimize the difference between the position matrix and rotation matrix of the target and actual positions, the target function is initially constructed, as shown in Equation 4:


$$\begin{array}{c} \min:Function\_1=\|[R_{x},R_{y},R_{z}]-\left[R_{x}^{e},R_{y}^{e},R_{z}^{e}\right]\|\\ Function\_2=\sqrt{{\left(P_{x}-P_{x}^{e}\right)}^{2}+{\left(P_{y}-P_{y}^{e}\right)}^{2}+{\left(P_{z}-P_{z}^{e}\right)}^{2}}\\ fitness=Function\_1+\alpha \cdot Function\_2 \end{array}$$ (4)

In Equation 4, *Function_l* represents the attitude error, calculated as the absolute error of the rotational angles along the three axes. *Function_2* represents the position error, calculated as the Euclidean distance between the three-dimensional coordinate points. Given the significant order-of-magnitude differences between these two errors, an adjustment factor *α* is introduced, where *α = 0.03*. Additionally, the redundancy of the robotic arm can lead to multiple inverse solutions for the same target point, which may reduce solving efficiency and cause singular positions. Furthermore, to improve solution efficiency and avoid singularities, a “soft and smooth” criterion is proposed, which aims to minimize the changes in joint angles. Based on this, the objective function is modified, as shown in Equation 5:


$$\begin{array}{c} fitness=Function\_1+\alpha \cdot Function\_2+\delta \cdot \sum {\mathrm{\backslash nolimits}}_{i=1}^{6}\left(\theta_{i+1}-\theta_{i}\right)\\ \delta =\frac{1}{\sum {\mathrm{\backslash nolimits}}_{i=1}^{6}\left(ub_{i}-lb_{i}\right)} \end{array}$$ (5)

In Equation 5, *lb* and *ub* represent the lower and upper rotational limits of the robotic arm joints, respectively.

### 2.3 Secretary bird optimization algorithm

Secretary Bird Optimization Algorithm (SOBA) [37], proposed by Youfa Fu et al. in 2024, is a bio-inspired optimization algorithm that mimics the survival strategies of secretary birds in their natural environment. The secretary bird, an African raptor renowned for its unique snake-hunting behavior, primarily feeds on reptiles in the African savannah. When catching snakes, the bird uses its physical advantage to monitor the snake’s movements from above, controlling the prey while circling, jumping, and attacking to exhaust the snake’s energy. Once the snake is weakened, the bird avoids direct confrontation by moving to its back and delivering a fatal blow with its sharp claws. SOBA is a stochastic search bio-heuristic optimization algorithm that models this behavior mathematically. The algorithm begins by randomly initializing a population to determine the initial values of the optimization problem. It evaluates the quality of population variables using the objective function and iteratively guides the population toward the optimal solution through its mathematical framework. The core of SOBA consists of two main strategies: the predation strategy and the escape strategy, which correspond to the secretary bird’s hunting and avoidance behaviors.

(1) Predation Strategy

During hunting, the behavior of secretary birds can be divided into three phases: searching for prey, exhausting the prey’s energy, and attacking the prey. SOBA replicates these phases mathematically by setting the population position (decision variable) as *β*, the dimension of the decision variable as *dim*, the maximum number of iterations as *Max_iteration*, and the current iteration as *Iteration*. The iterative process is divided into three phases, with the population position updated using Equations 6, 7, and 8.


$$IF:Iteration\leq \frac{1}{3}Max\_iteration,\beta_{i,j}^{new}=\beta_{i,j}+\gamma \cdot (\beta_{\tau 1}-\beta_{\tau 2})$$ (6)

$\beta_{i,j}$ is the value of the i-th individual in the j-th variable dimension. *γ* is a 1 × $\beta_{\tau 1},\beta_{\tau 2}$ are two randomly selected individuals from the population.


$$\begin{array}{c} IF:\frac{1}{3}Max\_iteration<Iteration\leq \frac{2}{3}Max\_iteration\\ \beta_{i,j}^{new}=\beta_{best}+e^{c^{4}}\cdot (\delta -0.5)\cdot (\beta_{best}-\beta_{i,j})\\ c=\frac{Iteration}{Max\_iteration} \end{array}$$ (7)

*β*_*best*_ represents the current best individual in the population. *e* is the natural logarithm. *δ* is a 1 × dim random array that follows a standard normal distribution.


$$\begin{array}{c} IF:Iteration>\frac{2}{3}Max\_iteration\\ \beta_{i,j}^{new}=\beta_{best}+(1-c{)}^{2\cdot c}\cdot \beta_{i,j}\cdot \varphi \end{array}$$ (8)

The weighted Lévy flight strategy, represented by *φ*, is used during the hunting process and can be calculated using Equation 9. *Γ* is the gamma function. *r*_*1*_ and *r*_*2*_ are random numbers in the range [0,1].


$$\begin{array}{c} \varphi =0.5\cdot Levy(\dim)\\ Levy(\dim)=0.01\cdot \frac{r_{1}\cdot \omega }{{\left|r_{2}\right|}^{0.667}}\\ \omega ={\left[0.05479\cdot \frac{\Gamma (2.5)}{\Gamma (1.25)}\right]}^{0.667} \end{array}$$ (9)

(2) Escape Strategy

The natural enemies of secretary birds include large predators such as hawks and foxes. When faced with these threats, secretary birds employ two main defense strategies: either they fly or run quickly to escape predators, or they use their environment for camouflage, making it difficult for predators to detect them. SOBA simulates these defensive behaviors using a mathematical model that updates the population’s location through Equation 10.


$$\left\{ \begin{array}{c} \beta_{i,j}^{new}=\beta_{best}+(2\cdot \delta -1)\cdot (1-c{)}^{2}\cdot \beta_{i,j},IF:rand<0.5\\ \beta_{i,j}^{new}=\beta_{i,j}+\mu \cdot (\beta_{\tau 1}-\eta \cdot \beta_{i,j}),ELSE \end{array}\right.$$ (10)

In Equation 10, *μ* is a 1 × dim random array of numbers that follows a normal distribution, while *η* is a randomly selected value between 1 and 2, which can be calculated using Equation 11. The function *round* is a rounding function applied to generate discrete values.


η=round[1+rand(1,1)] (11)

**Fig 4:**
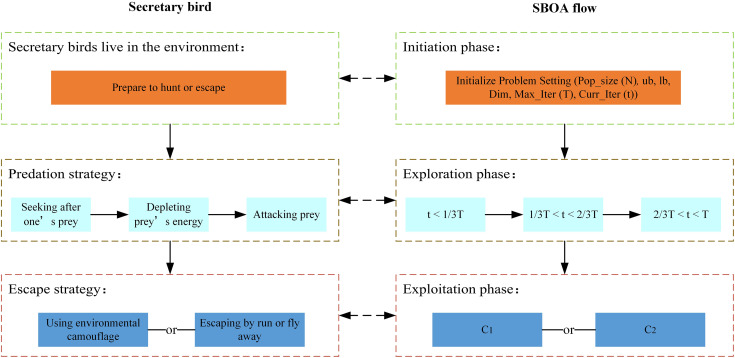
Flowchart of SBOA algorithm.

In summary, the flowchart of the SOBA algorithm is shown in Fig 4.

## 3. Improvement secretary bird optimization algorithm (ISBOA)

### 3.1 Oppositional variational perturbation strategy

The SBOA algorithm uses three methods to progressively search for food during the hunting phase, using time-based control. For example, during the initial stage of the iteration process, the prey search strategy is applied to update the population position. Similarly, the total iteration duration is divided into three phases, with different strategies employed in each phase. Guided by mathematical formulas, the numerical variables in the optimization problem are continuously updated with the goal of pursuing the optimal value of the objective function.

However, this approach of updating the population position using a single formula within a fixed phase has notable limitations. Specifically, it limits the diversity of population positions by neglecting concentrated regions of the search space and failing to fully explore the solution space. This can degrade the quality of solutions and reduce the algorithm’s performance. Enhancing solution quality is a common strategy to increase population diversity and improve optimization performance. Oppositional learning, as an effective approach, can improve the quality of population individuals and enable broader exploration of the solution space by computing the inverse solutions of individuals within the search space. In oppositional learning, the center point of the search boundary acts as the axis for calculating the inverse position of an individual. The mathematical expression for this calculation is shown in Equation 12:


β′=lower+upper−β (12)

*β* represents the current solution. *β′* represents the reverse solution. *upper* and *lower* denote the upper and lower boundary constraints of the optimization variables, respectively. As seen in Equation 12, oppositional learning tends to map solutions to relative regions of the search space. However, this can lead to solutions deviating from the central region, potentially causing the algorithm to avoid the optimal solution in the core region and wasting computational resources. To address this issue, this paper proposes a variation learning mechanism based on the oppositional learning strategy and integrates it into the search phase of SBOA. This mechanism expands the algorithm’s search area through population perturbation, thereby increasing the likelihood of identifying optimal solutions. The mathematical expression for the variation learning mechanism is provided in Equation 13:


$$\begin{array}{c} \begin{array}{c} \beta^{\prime \prime }=\left\{ \begin{array}{c} \tau_{1}\cdot (\beta^{\prime }-cp)+cp,\beta^{\prime }>cp\\ \tau_{1}\cdot (cp-\beta^{\prime })+cp,\beta^{\prime }\leq cp \end{array}\right.\\ cp=\frac{lower+upper}{2} \end{array} \end{array}$$ (13)

**Fig 5:**
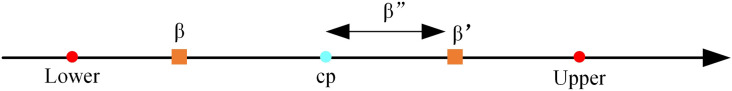
Oppositional variational perturbation strategy.

In Equation 13, *cp* represents the center point of the constraint boundary, and *τ*_*1*_ is a random number between [0,1]. A schematic diagram of the oppositional variational perturbation strategy is presented in Fig 5.

### 3.2 Golden sine development strategy

The SBOA algorithm exhibits weak robustness in solving optimization problems due to its simple structure and limited number of control parameters. This limitation becomes particularly evident in engineering problems, such as the inverse kinematics of robotic arms, where SBOA converges slowly within the solution space, making it challenging to find the optimal solution quickly and accurately. To address this issue, this paper introduces a golden sine development strategy aimed at accelerating the convergence speed of SBOA.


$$\beta_{i,j}^{new}=\beta_{i,j}\cdot \left|\sin(\delta_{1})\right|-\delta_{2}\cdot \sin(\delta_{1})\cdot \left|c_{1}\cdot \beta_{best}-c_{2}\cdot \beta_{i,j}\right|$$ (14)

The golden sine strategy incorporates the golden section method into the sine function, enabling the current solution to approach the target solution more efficiently. Its mathematical expression is shown in Equation 14, where *δ*_*1*_ and *δ*_*2*_ are two random numbers, $\delta_{1}\in [0,2\pi ],\delta_{2}\in [0,\pi ]$. The parameters *c*_*1*_*、c*_*2*_ are calculated as shown in Equation 15, with *L=−π* and *M = π*.


$$\begin{array}{c} c_{1}=(1-gold)\cdot (M-L)+L\\ c_{2}=gold\cdot (M-L)+L\\ gold=\frac{1-\sqrt{5}}{2} \end{array}$$ (15)

**Fig 6:**
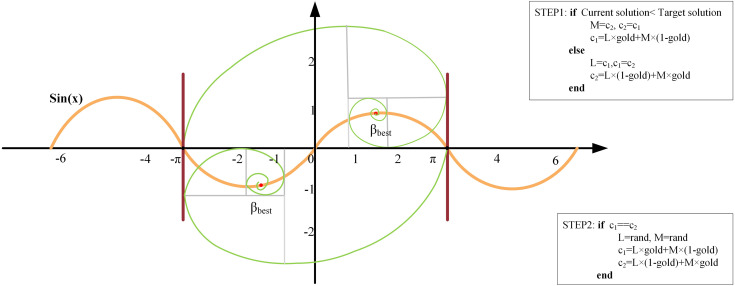
Golden sine development strategy.

Fig 6 illustrates the specific operational form of the golden sine development strategy. During the iterative process of SBOA, the partitioning method is utilized to approximate the target solution. This approach makes the solution update more efficient and significantly improves the algorithm’s convergence speed. The improved golden sine method not only enhances the quality of solutions but also improves the algorithm’s performance when dealing with complex optimization problems. By integrating this strategy, SBOA becomes more robust and effective in achieving accurate and efficient optimization results.

### 3.3 Evolutionary strategy

In the escape phase, the secretary bird decides whether to adopt the stochastic strategy of camouflage or escape based on whether a randomly generated value is greater than 0.5. However, in practical engineering applications, the complexity of optimization problems often causes some individuals within the population to fall into local optima, making it difficult for them to escape or converge effectively. This results in wasted computational resources and reduces the algorithm’s ability to escape local optimal solutions. To address this issue, this paper introduces an evolutionary strategy, which mimics the successive cross-evolution of biological genes, enabling multi-objective individuals to escape from local optima efficiently.

The evolutionary strategy shares similarities with the concept of evolutionary planning but was developed independently of genetic algorithms and evolutionary planning in Europe. Its basic concept and implementation process can be summarized as follows: ①The primary objective is to find the n-dimensional real vector *x*$F(x):R^{n}\to R$ which is associated with the extreme value of the function. ②Parent vectors are randomly selected from the defined search space, ensuring that initialization distributions are consistent across the population. ③Ch$x_{i}^{\prime }$ are generated by adding a zero-mean, Gaussian-distributed random variable (with a standard deviation pre-selected to match the standard deviation of *x*) to the parent vector. This process is repeated for each parent vector *x*_*i*_ (i = 1, …, P). ④T$F(x_{i}^{\prime })$, and the *P* vectors with the smallest errors are selected as the parent vectors for the next generation. ⑤New experimental data is continuously generated, and the vector with the smallest error is selected. This process continues until a satisfactory solution is found or the computation reaches its predefined limit.

**Fig 7:**
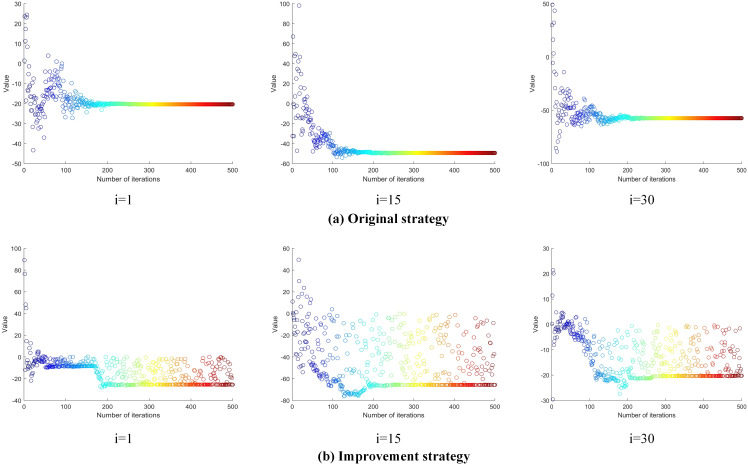
Evolutionary strategy effect diagram.

The evolutionary strategy is designed to accelerate population search and explore a broader solution space, improving the ability to escape local optimal solutions. To demonstrate its effectiveness, we evaluate it using the 30-dimensional CEC2017 function F1 as an example. The number of populations is set to 30, and the maximum number of iterations is set to 500. Fig 7 illustrates the changes in fitness values for the 1st, 15th, and 30th individuals in the population. In the original strategy, the initial fitness value of the first individual is relatively high and decreases rapidly as the number of iterations increases. However, the decreasing trend becomes smooth early on, indicating premature convergence to a local optimum. This suggests that the original strategy lacks sufficient global exploration capability. In contrast, under the evolutionary strategy, the first individual’s fitness value shows a much larger decline and exhibits stochastic fluctuations, maintaining population diversity. As a result, the fitness values are significantly lower compared to the original strategy, demonstrating superior optimization performance. For the 15th individual, the original strategy stabilizes its fitness value after approximately 100 iterations, indicating convergence. Although the objective value decreases, the premature convergence highlights the algorithm’s tendency to fall into local optima, limiting further optimization potential. On the other hand, the evolutionary strategy demonstrates robust exploration ability. Even in the middle stages (e.g., after 200 iterations), it can jump out of local optima and continue searching for better solutions. For the 30th individual, the original strategy falls into a local trap after about 200 iterations. At this point, the positions of the population individuals stagnate, making further optimization difficult. In contrast, the evolutionary strategy maintains dynamic population changes throughout the iterations. Consequently, the final objective function value achieved by the evolutionary strategy is significantly better than that of the original strategy. This highlights the evolutionary strategy’s superior global search ability and faster convergence toward the global optimum.

In summary, in order to overcome the limitations of the original SBOA algorithm, this paper combines the following three strategies to improve the algorithm and form an improved optimization algorithm ISBOA: in the early stage of the search, the positions of the population individuals are perturbed by the opposing variations in order to explore a wider solution space and improve the diversity of the population. In the middle stage of the search, the convergence process of population individuals is accelerated by the golden sine strategy, which significantly improves the optimization efficiency. In the late stage of search, the global search ability of the population is enhanced by cross-evolution to get rid of local traps and further optimize the positions of population individuals. Based on the improvement of the above three strategies, improved secretary bird optimization algorithm (ISBOA) is formed, and its pseudo-code is shown in Algorithm 1.

Algorithm 1 Pseudo code for ISBOA

*BEGINING*

*Inputs:* ***N_all, Max_ iteration, lower, upper***

*Initialization the individuals* ***β***_***i***_ *and calculate fitness*

*While* ***Iteration ≤ Max_ iteration***

 *For (i**** = 1,2……N_all****) do*

  *If* ***Iteration ≤ 1/3 Max_ iteration*** *do*

   *Adjust this place of Secretary bird using eq. (6)*

  *Elseif* ***1/3 Max_ iteration < Iteration ≤ 2/3 Max_ iteration***

   *Adjust this place of Secretary bird using eq. (7)*

  *Else*

   *Adjust this place of Secretary bird using eq. (8)*

  *End if*

  *Calculate the inverse population solution using eq. (13)*

  *Calculate fitness and select the best population individual*

 *End for*

 *For (i**** = 1,2……N_all****) do*

  *If* ***rand<0.5***

   *Adjust this place of Secretary bird using eq. (14)*

  *Else*

   *Adjust this place of Secretary bird using Evolutionary strategy*

  *End if*

  *Calculate fitness and select the best population individual*

 *End for*

*End while*

*Return the* ***best population individual*** *and* ***best score***

*END*

**Fig 8:**
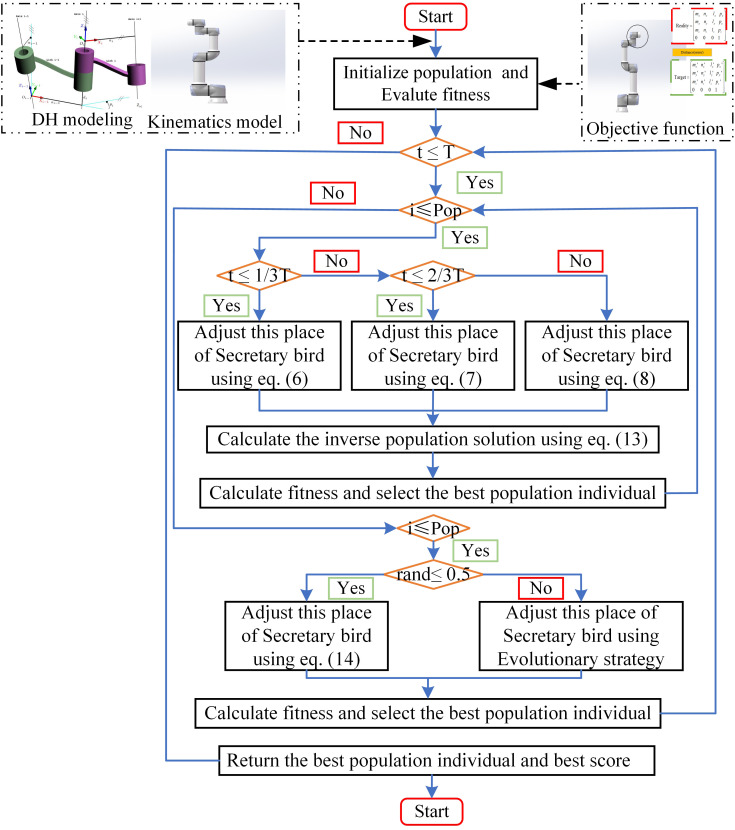
Inverse kinematics solution model based on ISBOA.

With the improved optimization capabilities of SBOA achieved through the enhancements discussed above, this paper develops an ISBOA-based inverse kinematics solution model for robotic arms using the objective function described in Section 2.2. The workflow of the proposed model is illustrated in Fig 8.

### 3.4 Algorithm complexity analysis

The computational complexity of an algorithm is a critical metric for evaluating its runtime performance, which can be analyzed based on the algorithm’s structure and implementation. In this paper, the Big O notation is applied to analyze the time complexity of ISBOA. Let *N* denote the size of secretary bird population, *D* denote the number of dimensions, and *T* denote the maximum number of iterations. The computational complexity of SBOA can be divided into three parts: O(population initialization), O(updating population position), and O(adaptation calculation). According to the literature, the computational complexity of SBOA is shown in Equation 16:


O(SBOA)=O(N)+O(T×N×D)+O(T×N)=O(N+TND+TN) (16)

The computational complexity of ISBOA proposed in this paper is as in Equation 17:


$$O(ISBOA)=O(N)+O(T\times N\times D)+O(T\times N^{2})=O(N+TND+TN^{2})$$ (17)

## 4. Experimental results and analysis

The experimental part is set up with three groups of different categories of experiments, which are numerical experiments, simulation experiments and physical verification. Firstly, the well-known CEC2017 test function is used in the numerical experiments to prove the success of ISBOA and the effectiveness of the improvement strategy. Then in order to verify the feasibility of the proposed inverse kinematics solving model, 4, 6, and 7-DOF robotic arms are used, respectively, for the inverse kinematics solving simulation experiments. Finally, based on the inverse kinematics solution method of ISBOA, for the pyrotechnic grain assembly, CoppeliaSim software is firstly used for the simulation verification, and then the solid robotic arm UR16e is used for the experiments. The control groups configured in the numerical and simulation experiments used well-known optimization algorithms: Particle Swarm Optimization (PSO) [38], Slime Mould Algorithm (SMA) [39], Sand Cat Swarm Optimization (SCSO) [40], Crayfish Optimization Algorithm (COA) [41], Walrus Optimizer (WO) [42], Black-winged Kite Algorithm (BKA) [43], SBOA, and ISBOA. The parameter settings of these eight optimization algorithms, including this new ISBOA algorithm, are given in Table 2 below.

**Table 2:** Optimization algorithm parameter settings.

Comparison algorithm	Time/Year	Parameter settings	Individual number	Maximum iteration
PSO [44]	1995	W = 0.9; m = 1.4946; n = 1.4946	30	500
SMA [39]	2020	z = 0.03	30	30, 1000
SCSO [40]	2023	s = 2	30	500
COA [37]	2023	Temp=[20,35],	30	500
WO [42]	2024	P = 0.4;	100	2000
BKA [43]	2024	p = 0.9;	30	500
SBOA [37]	2024	\	30	500
ISBOA	\	\	\	\

### 4.1 Numerical experiments

**Fig 9:**
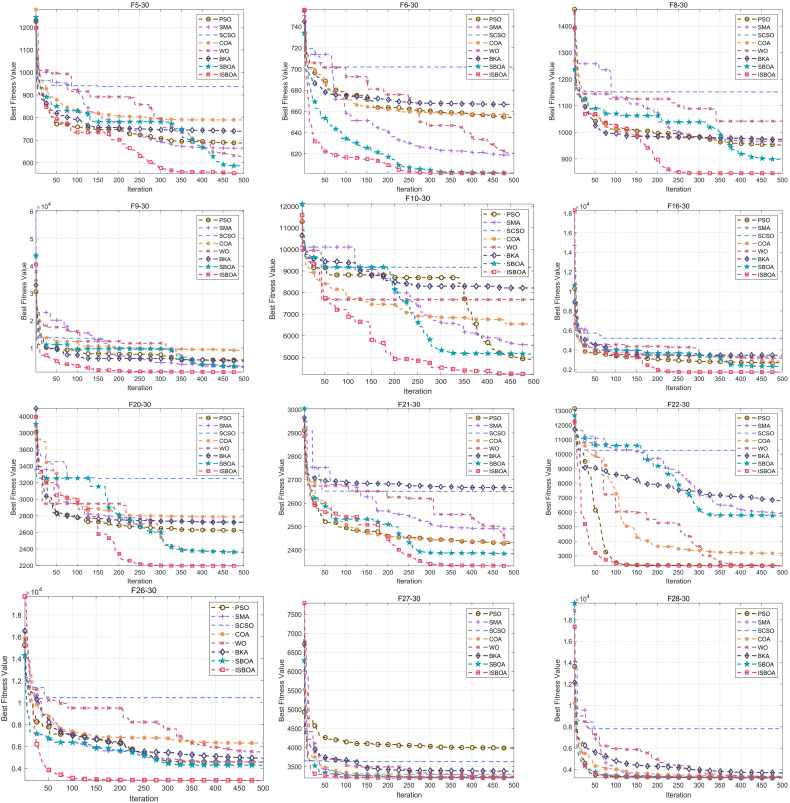
CEC2017 convergence curve.

The numerical experiments are conducted using the CEC2017 test set with variable dimensions set to 30. The performance of ISBOA is compared to seven other optimization algorithms: PSO, SMA, SCSO, COA, WO, BKA, and SBOA. The population size for all algorithms is set to 30, the maximum number of iterations is limited to 500, and each experiment is repeated 30 times for statistical analysis [45–47]. The experiments are implemented on a 64-bit Windows platform using MATLAB 2023a, running on an i7-12700F processor. Fig 9 illustrates the convergence curves for several functions.

From the subplots in Fig 9, it is evident that ISBOA demonstrates a significantly faster convergence speed compared to other algorithms on most functions. Notably, for functions such as F5, F8, F10, and F22, ISBOA achieves low fitness values that are difficult for other algorithms to approach, even within fewer iterations. This rapid convergence is attributed to the oppositional variational perturbation strategy, which extends the algorithm’s search area by perturbing the population, increasing population diversity, and enhancing global search capability. These improvements enable ISBOA to quickly approach the optimal solution. For functions such as F16, F20, F26, and F28, ISBOA achieves significantly lower final fitness values compared to other algorithms. This improvement is due to the golden sine development strategy, which accelerates convergence and avoids premature stagnation caused by rapid convergence. By effectively balancing exploration and exploitation during the development stage, this strategy allows ISBOA to find more accurate solutions. In complex functions (e.g., F10, F21, F26), PSO, WOA and SMA are prone to falling into local optima, as evidenced by flattened or minimally fluctuating convergence curves. In contrast, ISBOA utilizes an evolutionary strategy that simulates the continuous cross-evolution of biological genes, enabling population individuals to conduct a wider search in the local optimal region. This strategy effectively allows ISBOA to escape local optima, enhance convergence efficiency, and exhibit a continuous decline in fitness values. Consequently, ISBOA consistently outperforms other algorithms, demonstrating its ability to find better solutions even in highly complex optimization problems.

Table 3 presents the statistical results for the CEC2017 test set (only F1-F10 are displayed for clarity and conciseness), including the mean, standard deviation, median, and maximum (or minimum for extreme value optimization problems). The bolded values represent the current optimal results. From Table 3, it can be seen that for the average of 30 fitness values, ISBOA wins in F1, F2, F3, F4, F6, F7, and F10. Meanwhile, the average value reflects the overall performance of the algorithm on this test set. Experimental data show that ISBOA, with its excellent global search capability, makes the population individuals traverse the search space and finally obtains a smaller fitness mean value on the CEC2017 test set. For F5, F8, and F9, although ISBOA does not achieve the best results, its performance is very close to the optimal algorithms, indicating broad applicability and consistent performance. ISBOA achieves the smallest standard deviations on functions F1, F2, and F6, signifying high stability on these functions. Even for functions where it does not achieve the optimal results (e.g., F3 and F4), ISBOA maintains low standard deviations, indicating reliability and suitability for solving complex optimization problems. ISBOA demonstrates the best median values for functions F2, F3, F6, and F10, further validating its superior performance on these functions. The consistency between the median and mean results highlights ISBOA’s ability to consistently find better solutions across runs, showing robust performance even in typical runs. ISBOA achieves the best worst-case performance on functions F1, F3, F6, and F10, demonstrating its ability to maintain high-quality solutions even in unfavorable scenarios. This robust performance is attributed to ISBOA’s three improvement strategies—oppositional variational perturbation, golden sine development, and evolutionary strategy—which enhance its ability to escape local optima and maintain competitiveness across diverse scenarios.

**Table 3:** CEC2017 statistics.

		PSO	SMA	SCSO	COA	WO	BKA	SBOA	ISBOA
F1	Ave	2.9810E + 06	1.2028E + 05	3.9879E + 10	1.0561E + 09	1.6311E + 08	9.4857E + 09	8.0034E + 04	**2.7612E + 04**
	Std	1.7193E + 06	5.1228E + 04	3.0220E + 09	9.9076E + 08	1.2098E + 08	8.7109E + 09	3.2431E + 05	**1.9685E + 04**
	Med	2.2319E + 04	1.0316E + 05	3.9753E + 10	8.2186E + 08	1.2583E + 08	6.9647E + 09	**1.9751E + 04**	2.5031E + 06
	Worst	8.0930E + 06	2.4526E + 05	4.7706E + 10	4.5346E + 09	5.4537E + 08	4.1575E + 10	1.7946E + 06	**8.1137E + 04**
F2	Ave	1.1602E + 18	6.0579E + 14	2.9914E + 43	1.2687E + 29	2.0181E + 26	1.0978E + 40	4.0507E + 17	**1.2383E + 13**
	Std	5.2750E + 18	2.1567E + 15	1.2321E + 44	6.9419E + 29	7.1155E + 26	6.0104E + 40	8.9998E + 17	**2.5470E + 13**
	Med	2.2108E + 15	6.6637E + 13	6.4880E + 38	5.4604E + 22	5.0840E + 23	1.4151E + 26	1.2172E + 16	**2.2029E + 12**
	Worst	2.9010E + 19	1.1900E + 16	6.3234E + 44	3.8024E + 30	3.6603E + 27	3.2921E + 41	4.0481E + 18	**1.1680E + 14**
F3	Ave	4.5939E + 04	3.4701E + 04	8.3381E + 04	1.0962E + 05	6.3855E + 04	3.7406E + 04	2.4389E + 04	**2.2393E + 04**
	Std	7.3269E + 03	1.6613E + 04	8.6434E + 03	2.7319E + 04	8.5276E + 03	1.8909E + 04	**6.6830E + 03**	1.4883E + 04
	Med	4.2948E + 04	3.0711E + 04	8.6653E + 04	1.0227E + 05	6.5321E + 04	3.2603E + 04	2.3751E + 04	**2.3732E + 04**
	Worst	9.8056E + 04	8.2034E + 04	9.2873E + 04	1.7639E + 05	7.8544E + 04	8.6063E + 04	3.4819E + 04	**3.4627E + 04**
F4	Ave	5.0110E + 02	5.0909E + 02	1.1670E + 04	6.1047E + 02	5.8104E + 02	2.0215E + 03	5.0626E + 02	**4.7648E + 02**
	Std	2.7743E + 01	**2.1139E + 01**	3.7246E + 03	5.8615E + 01	6.0516E + 01	2.8228E + 03	3.0244E + 01	3.1095E + 01
	Med	5.1197E + 02	5.0339E + 02	1.1164E + 04	6.0134E + 02	5.5850E + 02	9.8628E + 02	5.1044E + 02	**4.8124E + 02**
	Worst	**5.3915E + 02**	5.5474E + 02	1.8352E + 04	7.4261E + 02	8.0319E + 02	1.4376E + 04	5.9795E + 02	5.5020E + 02
F5	Ave	6.0264E + 02	6.2935E + 02	9.2450E + 02	7.5374E + 02	6.7632E + 02	7.5730E + 02	**5.8362E + 02**	6.9222E + 02
	Std	2.8039E + 01	3.2240E + 01	2.0206E + 01	5.9617E + 01	6.8796E + 01	5.0275E + 01	**2.0204E + 01**	3.4180E + 01
	Med	5.9788E + 02	6.3202E + 02	9.2475E + 02	7.6835E + 02	6.5308E + 02	7.5623E + 02	**5.8533E + 02**	6.9924E + 02
	Worst	6.7013E + 02	7.1584E + 02	9.5382E + 02	8.2441E + 02	8.1633E + 02	9.3043E + 02	**6.1744E + 02**	7.4697E + 02
F6	Ave	6.4934E + 02	6.1620E + 02	6.9406E + 02	6.4912E + 02	6.3399E + 02	6.6103E + 02	6.0275E + 02	**6.0064E + 02**
	Std	7.5011E + 00	9.0437E + 00	7.1548E + 00	1.6384E + 01	1.6766E + 01	8.1742E + 00	3.2407E + 00	**4.9615E-01**
	Med	6.5020E + 02	6.1394E + 02	6.9450E + 02	6.5557E + 02	6.2938E + 02	6.5986E + 02	6.0183E + 02	**6.0050E + 02**
	Worst	6.6522E + 02	6.5255E + 02	7.0634E + 02	6.7230E + 02	6.9678E + 02	6.8573E + 02	6.1665E + 02	**6.0260E + 02**
F7	Ave	9.5605E + 02	8.9490E + 02	1.3779E + 03	1.2532E + 03	9.9129E + 02	1.2311E + 03	8.5380E + 02	**8.2655E + 02**
	Std	5.1149E + 01	4.8856E + 01	6.5464E + 01	9.3876E + 01	7.8523E + 01	8.1521E + 01	4.2324E + 01	**2.8900E + 01**
	Med	9.6047E + 02	8.8305E + 02	1.3719E + 03	1.2924E + 03	9.9251E + 02	1.2255E + 03	8.4403E + 02	**8.2040E + 02**
	Worst	1.0828E + 03	1.0182E + 03	1.4867E + 03	1.3649E + 03	1.1380E + 03	1.4286E + 03	9.8739E + 02	**8.9031E + 02**
F8	Ave	8.9277E + 02	9.3356E + 02	1.1305E + 03	9.8683E + 02	9.7353E + 02	9.7859E + 02	**8.7657E + 02**	9.4112E + 02
	Std	2.2191E + 01	2.9273E + 01	2.3769E + 01	1.9087E + 01	7.3814E + 01	4.4722E + 01	**1.4762E + 01**	2.6489E + 01
	Med	8.9365E + 02	9.3478E + 02	1.1276E + 03	9.9071E + 02	9.4624E + 02	9.7235E + 02	**8.7369E + 02**	9.4423E + 02
	Worst	9.4131E + 02	9.7898E + 02	1.1846E + 03	1.0245E + 03	1.1593E + 03	1.1442E + 03	**9.0880E + 02**	1.0267E + 03
F9	Ave	1.4747E + 03	4.6317E + 03	1.0661E + 04	7.2867E + 03	6.5442E + 03	5.8164E + 03	**1.4560E + 03**	6.7269E + 03
	Std	1.0187E + 03	1.7700E + 03	1.7565E + 03	1.9446E + 03	3.7982E + 03	1.5357E + 03	**5.2894E + 02**	1.7850E + 03
	Med	**9.8756E + 02**	4.6867E + 03	1.0813E + 04	7.2789E + 03	5.7299E + 03	5.5493E + 03	1.2980E + 03	6.3320E + 03
	Worst	5.6243E + 03	9.5266E + 03	1.3790E + 04	1.0551E + 04	1.3543E + 04	1.2960E + 04	1.0861E + 04	**2.7439E + 03**
F10	Ave	4.6146E + 03	4.7455E + 03	8.7692E + 03	6.0389E + 03	8.0222E + 03	5.7780E + 03	4.7886E + 03	**4.4709E + 03**
	Std	6.9011E + 02	7.8155E + 02	6.2920E + 02	8.0545E + 02	1.6915E + 03	1.1832E + 03	7.5885E + 02	**3.7736E + 02**
	Med	4.6508E + 03	4.7800E + 03	8.7875E + 03	6.0189E + 03	8.8592E + 03	5.5572E + 03	4.6306E + 03	**4.6257E + 03**
	Worst	5.8544E + 03	6.7895E + 03	9.4229E + 03	7.8742E + 03	9.4509E + 03	8.3498E + 03	5.9660E + 03	**5.5143E + 03**

**Fig 10:**
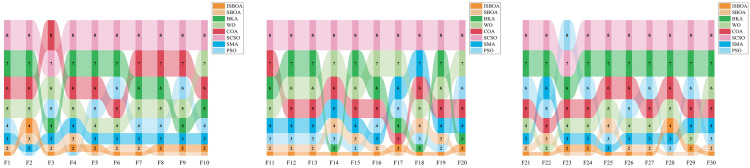
CEC 2017 algorithm ranking chart.

Fig 10 illustrates the ranking of the eight algorithms on the CEC2017 test functions. ISBOA ranks first in a total of 18 functions, outperforming other algorithms. From the convergence graphs and statistical tables presented in the numerical experiments, it is evident—both qualitatively and quantitatively—that ISBOA excels in convergence speed, convergence accuracy, and the ability to escape local optima compared to the original SBOA and other comparison algorithms. Its three major improvement strategies (oppositional variational perturbation, golden sine development, and evolutionary strategy) play key roles at different stages, so that the algorithm shows stronger adaptability to high-dimensional complex optimization problems, indicating that in numerical experiments, ISBOA has a certain degree of competitiveness with the optimization algorithms in recent years.

### 4.2 Simulation experiments

The numerical experiments in Section 4.1 demonstrate the effectiveness of ISBOA’s improvement strategies from a theoretical and mathematical perspective. In the manufacturing industry, the assembly of pyrotechnic grain serves as a critical application scenario due to its high complexity and inherent danger. This process requires exceptional assembly precision, strict safety standards. To address these specific requirements, this paper proposes an ISBOA-based inverse kinematics solver model in Section 3 to achieve accurate, and high-precision joint angle calculations for the assembly of pyrotechnic grain. To verify the feasibility of the proposed model, simulation experiments are conducted using robotic arms with 4, 6, and 7-DOF to solve the inverse kinematics problem.

Firstly, based on the DH parameter table of the robotic arms, the Monte Carlo method is applied to visualize and analyze the working domain of the robotic arms, and to set up target points reachable by the arms. Subsequently, seven optimization algorithms—PSO, SMA, SCSO, COA, WO, BKA, and SBOA—are used for comparison. The experiments are conducted with a population size of 30, a maximum of 500 iterations, and 30 independent runs for statistical analysis. The experiments are implemented on a 64-bit Windows platform using MATLAB 2023a on an i7-12700F processor.

#### 4.2.1 4-DOF tandem robotic arm.

**Fig 11:**
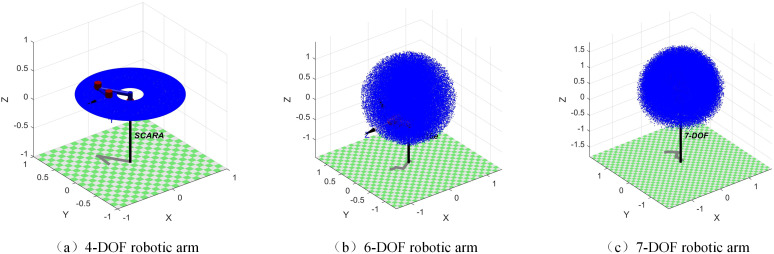
Robotic arm working space.

Table 4 provides the DH parameter list for the 4-DOF SCARA robotic arm. *a* is the joint angle, *b* is the joint distance, *c* is the length of the connecting rod, and *d* is the torsion angle of the connecting rod. The working domain of the robotic arm is analyzed using the Monte Carlo method, as illustrated in Fig 11(a). Based on this analysis, the objective point is set to (0.56, −0.31, 0.20). Among the four joints, the joints four are the moving joints, and the rest are the rotary joints.

**Table 4:** DH parameter list of SCARA.

No. Joint	*a (rad)*	*b (m)*	*c (m)*	*d (rad)*	Joint angle boundary (rad) Linear displacement (m)	Target point
1	0	0.2	0.5	0	[-3.14,3.14]	(0.56, −0.31, 0.20)
2	0	0	0.3	0	[-3.14,3.14]	
3	0	0	0	0	[-3.14,3.14]	
4	0	0	0	-π/2	[0, 0.5]	

**Fig 12:**
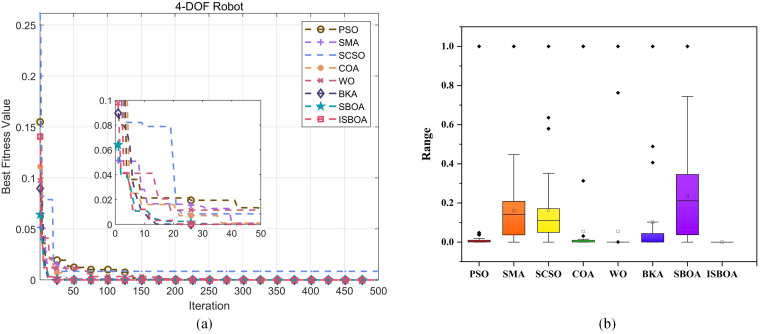
Inverse solution of convergence curve and fitness of 4-DOF robotic arm.

Fig 12(a) shows the convergence curves of eight optimization algorithms in solving the inverse kinematics problem of the SCARA robotic arm. From the figure, it is evident that most algorithms converge quickly within the first 50 iterations. ISBOA, however, achieves convergence to 2.49 × 10^−4^ in less than 30 iterations, exhibiting the fastest convergence rate and outperforming all other algorithms. In contrast, PSO and SMA converge more slowly, reaching stability after 50 iterations with fitness values of 1.2 × 10^−2^ and 1.1 × 10^−2^, respectively. The ISBOA curve is closest to 0 and demonstrates the lowest fitness value, indicating superior solution accuracy. After 50 iterations, the fitness values of BKA and SBOA approach ISBOA but remain slightly inferior in both convergence speed and accuracy. Algorithms such as SCSO, COA, and WO exhibit higher fitness values, indicating poorer accuracy in solving this problem. PSO and SMA perform moderately well, with better convergence speed and accuracy than SCSO and COA, but fall short of ISBOA’s performance.

After 30 cycles of computation, the best fitness values achieved by the algorithms are recorded. Due to the small scale of the fitness values, normalization is applied to process the data, and the results are visualized as box-and-line plots in Fig 12(b). The ISBOA algorithm shows the smallest and most concentrated distribution of bins, indicating the best fitness distribution, minimal fluctuation range over 30 cycles, and high stability with no outliers. The WO algorithm also exhibits minimal bins but has outliers, resulting in a larger average. For PSO, COA, and BKA, the box area is relatively small, and while outliers are present, they are closer to the box, indicating small data fluctuations and good accuracy, though stability is slightly worse. Conversely, SMA, SCSO, and SBOA show higher boxes with larger distribution ranges, indicating poorer fitness values and significantly lower solution quality compared to the other algorithms. The presence of numerous outliers and a wide fluctuation range further highlights the limited effectiveness and instability of these algorithms in solving this problem.

**Fig 13:**
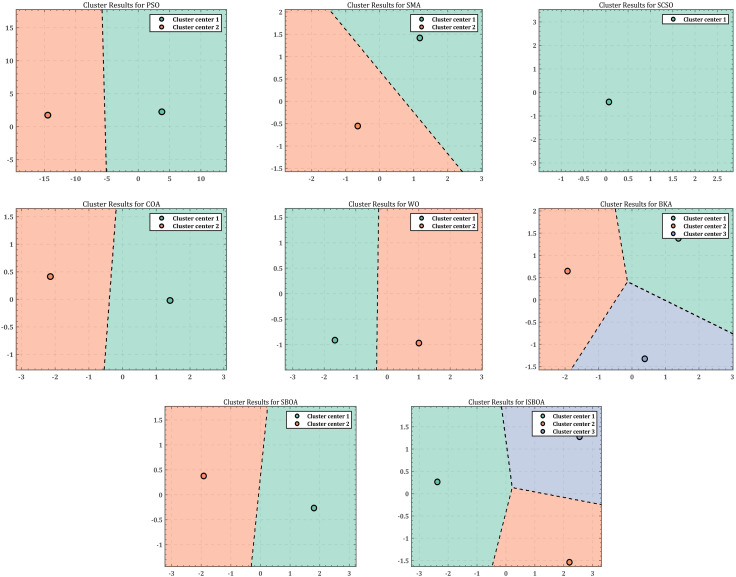
Clustering results of inverse solution of 4-DOF robotic arm.

As the redundancy of the robotic arm increases, solving the inverse kinematic joint angles for a single target point often yields different results, highlighting the diversity of inverse solutions. To evaluate the diversity of inverse solutions, the results of the eight optimization algorithms are recorded 30 times. The data are then downscaled using Principal Component Analysis (PCA) and clustered using K-means to analyze the performance of ISBOA in terms of inverse solution diversity. Fig 13 presents the clustering results of the optimization algorithms when solving the inverse solution joint angles of the SCARA robotic arm. In the clustering analysis of inverse solutions, PSO and SMA both exhibit two clustered regions separated by straight lines. However, PSO shows a more symmetric distribution of cluster centers, whereas SMA is skewed to one side, indicating limited diversity in its inverse solutions. SCSO performs poorly, with all its inverse solutions concentrated in a single region, completely lacking diversity. COA, while also displaying two clustered regions, shows slightly better diversity compared to SCSO, but its search diversity remains limited. The inverse solution results of WO are distributed across two symmetric regions, demonstrating moderate diversity. In contrast, BKA and ISBOA perform significantly better. BKA forms three clustered regions with complex boundaries, indicating enhanced diversity in its inverse solutions. ISBOA demonstrates the highest diversity of inverse solutions, with nonlinearly separated boundaries and uniformly distributed cluster centers. This result highlights ISBOA’s superior exploration capability, as it fully utilizes the search space to find a variety of solutions. Although SBOA is also divided into two cluster regions, the diversity of its inverse solutions is limited, and some regions are not fully explored.

**Fig 14:**
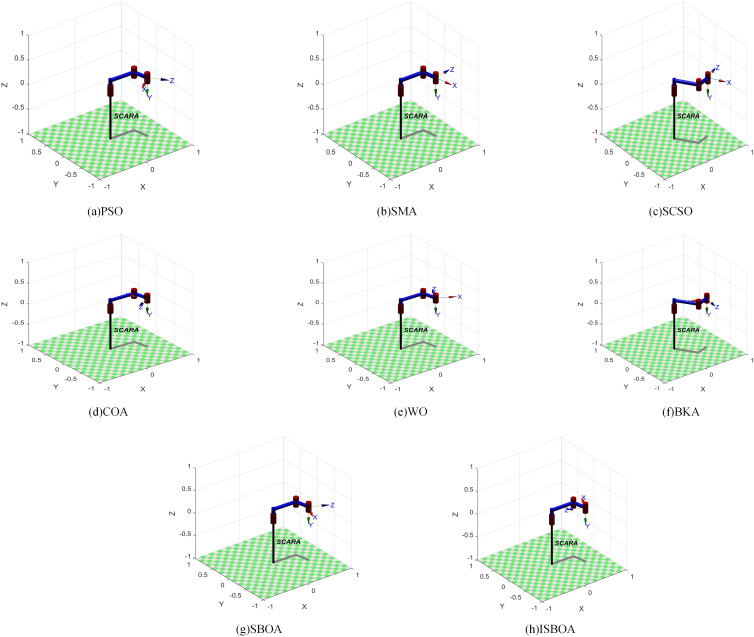
Visualization results of inverse solution of 4-DOF robotic arm.

Table 5 displays the results of ISBOA and the other seven optimization algorithms for solving the inverse kinematics of the SCARA robotic arm. The penultimate column represents the fitness values (with bold text indicating the optimal value). It can be known from Table 5 that both ISBOA and BKA achieve the minimum fitness value of 0. The shortest running time (3.4089s) is the SCSO algorithm, when the running time of ISBOA is slightly longer than that of SCSO. Running time reflects the consumption of computing resources and it is often difficult to balance between accuracy and efficiency. Given that the motion position of the robotic arm in precision assembly must be accurate, it is feasible to appropriately sacrifice the runtime to obtain the best accuracy. Both ISBOA and BKA successfully reach the target point with an error of zero. These results are visualized using the Robotics Toolbox in MATLAB, as shown in Fig 14.

**Table 5:** 4-DOF robotic arm joint angle.

Optimization algorithm	Joint angle(rad) or Linear displacement (m)				Error	Running time (s)
PSO	−0.03	−1.34	−2.23	1.14	3.2994E-12	3.4195
SMA	−0.03	−1.34	0.00	0.00	2.2616E-06	3.4613
SCSO	−0.96	1.25	−1.33	0.00	2.0347E-02	**3.4089**
COA	−0.03	−1.34	−3.14	0.34	7.3120E-08	5.8573
WO	−0.03	−1.34	0.57	0.31	8.0486E-08	3.4991
BKA	−0.98	1.34	2.29	0.26	**0**	6.8388
SBOA	−0.03	−1.34	−0.67	0.00	5.5511E-17	7.2198
ISBOA	−0.03	−1.34	2.13	0.34	**0**	5.9719

By substituting the inverse solution joint angles of ISBOA from Table 5 into its homogeneous transformation matrix, the resulting position matrix confirms that the joint angles computed by ISBOA accurately reach the target point.


$$A_{0}^{4}=\left[\begin{array}{c} 0.4486\;\;\;\;\;\;0\;\;\;\;\;\;\;-0\mathrm{.8937}\;\;\;\;\;\;\;\;\;\;0.56\\ 0.8937\;\;\;\;\;\;0\;\;\;\;\;\;\;\;\;\;0.4486\;\;\;\;\;\;-0.31\\ 0\;\;\;\;\;\;\;\;\;\;\;-1\;\;\;\;\;\;\;\;\;\;\;\;\;\;\;0\;\;\;\;\;\;\;\;\;\;\;\;\;\;\;\;\;\;\;\;0.2\\ 0\;\;\;\;\;\;\;\;\;\;\;\;\;\;\;0\;\;\;\;\;\;\;\;\;\;\;\;\;\;0\;\;\;\;\;\;\;\;\;\;\;\;\;\;\;\;\;\;\;\;\;1 \end{array}\right]$$

#### 4.2.2 6-DOF tandem robotic arm.

Table 6 provides the DH parameter list for the 6-DOF UR16e robotic arm. The working domain of the robotic arm is analyzed using the Monte Carlo method, as illustrated in Fig 11(b). Based on this analysis, the target point is set to (0.02, 0.76, 0.14).

**Table 6:** DH parameter list of UR16e.

No. Joint	*a (rad)*	*b (m)*	*c (m)*	*d (rad)*	Joint angle boundary (rad)	Target point
1	0	0.1807	0	π/2	[-3.14,3.14]	(0.02, 0.76, 0.14)
2	0	0	−0.4784	0		
3	0	0	−0.36	0		
4	0	0.17415	0	π/2		
5	0	0.11985	0	-π/2		
6	0	0.11655	0	0		

**Fig 15:**
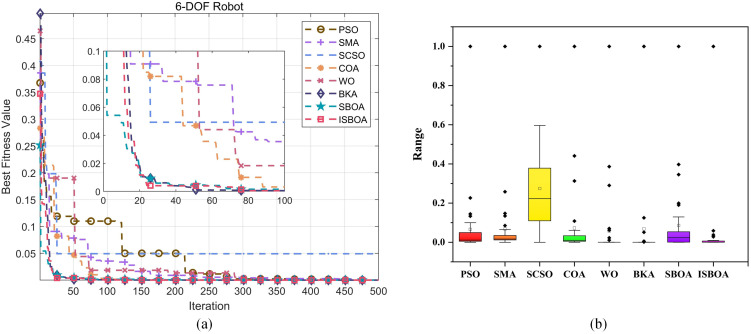
Inverse solution of convergence curve and fitness of 6-DOF robotic arm.

According to the convergence curve in Fig 15(a), ISBOA demonstrates excellent convergence speed and solution accuracy when compared to other algorithms. During the first 50 iterations, ISBOA quickly converges to 4.1 × 10^−3^ within the first 20 iterations, making it the fastest converging algorithm. SBOA and BKA, although following closely, are still slightly inferior to ISBOA. In contrast, SMA, PSO, and WO converge more slowly, requiring approximately 250 iterations to stabilize. SCSO and COA show the slowest convergence rates, maintaining high fitness values during the first 50 iterations. In the 50–500 iterations, ISBOA continues to outperform the other algorithms, eventually converging to a fitness value near zero, indicating the highest solution accuracy. While SBOA and BKA also achieve low fitness values, their accuracies remain slightly inferior to ISBOA. SMA and PSO show moderate fitness values, performing better than SCSO and COA, but still lagging behind ISBOA. Local zoom-in plots further confirm ISBOA’s early convergence superiority, as its curve drops rapidly within the first 50 iterations. Although the curves of BKA and SBOA are close to each other, their overall performance is slightly less optimal. By comparison, SCSO and COA exhibit minimal convergence in the early stage, further emphasizing ISBOA’s superiority in solving such problems.

The boxplot of the normalized data in Fig 15(b) further highlights ISBOA’s exceptional stability and solution quality. The algorithm exhibits the smallest and most centralized box, with almost no outliers, the smallest fluctuation range in fitness values, and the best solution quality across 30 experiments. In contrast, SBOA and BKA also demonstrate high accuracy with relatively small boxes and narrow distribution ranges, but they feature a few outliers, rendering their stability slightly inferior to ISBOA. PSO, SMA, and COA exhibit decreased stability and accuracy, with wider box distributions and more outliers. Among them, SCSO performs the worst, showing a high and wide box distribution, large fluctuations in fitness values, poor solution quality, and many outliers, indicating instability on this problem. The WO algorithm has a narrower box and occasionally achieves better solutions, but its overall performance is unstable and contains more outliers.

**Fig 16:**
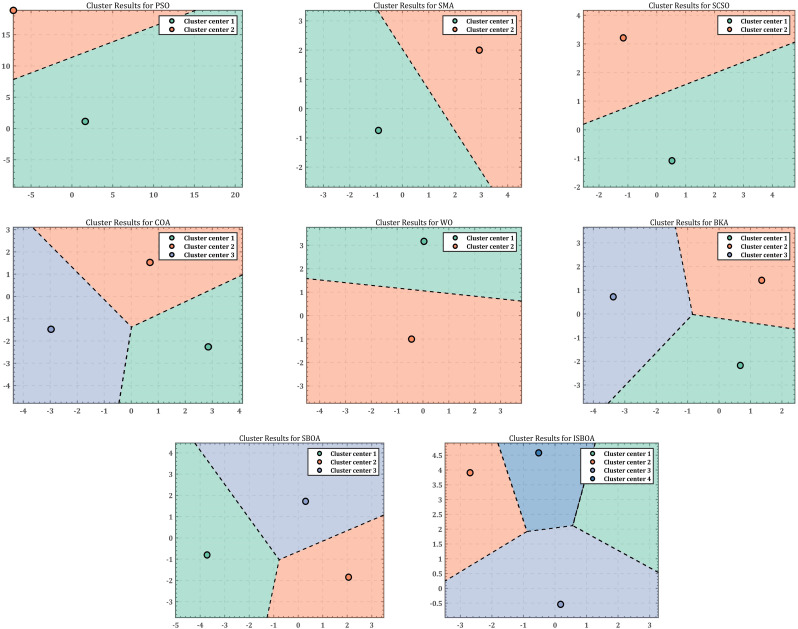
Clustering results of inverse solution of 6-DOF robotic arm.

In comparing the diversity of inverse solutions generated by eight optimization algorithms (PSO, SMA, SCSO, COA, WO, BKA, SBOA, and ISBOA) for solving the inverse kinematics problem of a 6-DOF robotic arm, cluster analysis is performed. The results are shown in Fig 16, where the inverse solutions of PSO, SMA, and SCSO are primarily concentrated in two simple, linearly separated clustering regions, demonstrating low diversity and limited exploration capabilities. In contrast, the COA algorithm forms three clustering regions with folded boundaries, indicating improved diversity in the inverse solutions. The WO algorithm exhibits a relatively balanced distribution of clustering centers, but the overall clustering region remains simple, and the diversity is still insufficient. The BKA algorithm generates three clustering regions with complex folded boundaries and uniformly distributed clustering centers, showcasing good inverse solution diversity. Similarly, the SBOA algorithm also forms three clustering regions with folded boundaries, but its distribution balance is slightly inferior to that of BKA. On the other hand, the ISBOA algorithm achieves the best performance, exhibiting four nonlinearly separated, complex clustering regions with uniformly distributed clustering centers. This highlights ISBOA’s ability to fully explore the solution space and effectively adapt to the complex inverse kinematics of highly redundant robotic arms.

**Fig 17:**
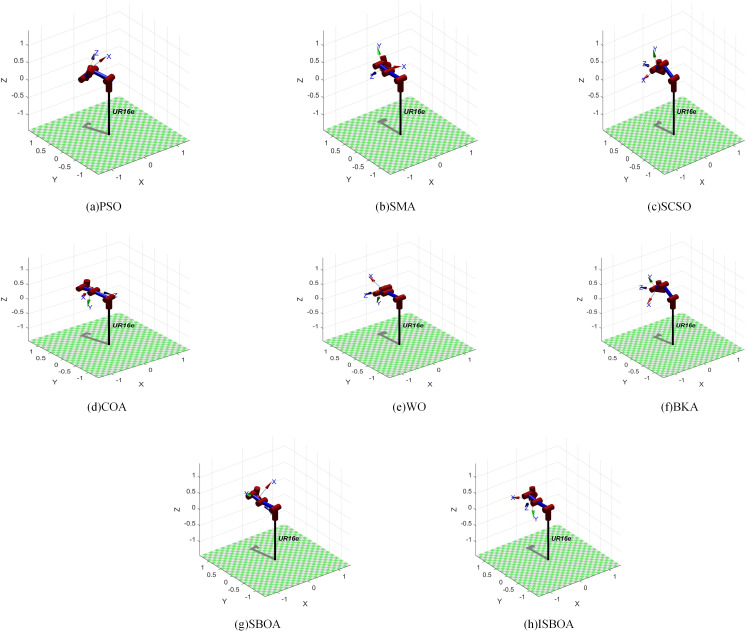
Visualization results of inverse solution of 6-DOF robotic arm.

The results of ISBOA and the other seven optimization algorithms for solving the inverse kinematics of the UR16e robotic arm are presented in Table 7, where the penultimate column lists the fitness values (with the optimal values bolded). As can be seen in Table 7, ISBOA has the best fitness value of 2.0935 × 10^-14^, followed by BKA, which has the best fitness value of 2.1250 × 10 ⁻ ¹⁴. Similarly, the computational efficiency of ISBOA is not the fastest and lags behind that of the top-ranked PSO (4.2171s). However, the optimal fitness value of ISBOA is nine orders of magnitude higher than that of PSO. The fitness value reflects the degree of difference between the predicted pose and the true pose after the optimization algorithm solves the joint angle. These results are visualized using the Robotics Toolbox in MATLAB, as shown in Fig 17.

**Table 7:** 6-DOF robotic arm joint angle.

Optimization algorithm	Joint angle(rad)						Error	Running time
PSO	1.92	3.34	−1.02	−5.00	0.45	−1.25	9.0406E-05	**4.2174**
SMA	1.63	−3.14	0.16	2.60	2.85	0.01	6.2518E-04	4.2741
SCSO	1.76	−2.56	−1.24	−2.62	−1.47	−0.10	7.0073E-02	4.3087
COA	1.82	−3.00	−0.09	−3.10	1.31	3.04	2.3720E-05	7.6364
WO	1.62	3.13	−0.40	0.88	3.10	0.11	5.6333E-06	4.4736
BKA	1.76	−2.43	−1.34	−2.72	−1.68	−0.47	2.1250E-14	8.9695
SBOA	1.74	−3.10	0.19	−3.14	1.80	0.98	3.8246E-12	8.8487
ISBOA	1.64	3.14	0.20	2.80	2.62	−3.14	**2.0935E-14**	8.1330

Finally, substituting the inverse-solved joint angles of ISBOA from Table 7 into its homogeneous transformation matrix yields the position matrix, confirming that the joint angles computed by ISBOA accurately reach the target point.


$$A_{0}^{6}=\left[\begin{array}{c} -0.5605\;\;\;\;\;\;\;\;\;0.0107\;\;\;\;\;\;\;-0\mathrm{.8281}\;\;\;\;\;\;\;\;\;\;0.02\\ \;\;\;0.8189\;\;\;\;\;\;\;-0.1413\;\;\;\;\;\;-0.5562\;\;\;\;\;\;\;\;0.76\\ -0.1230\;\;\;\;\;\;-0.9899\;\;\;\;\;\;\;\;\;\;0\mathrm{.0704}\;\;\;\;\;\;\;\;\;0.14\\ \;\;\;\;\;\;\;\;\;0\;\;\;\;\;\;\;\;\;\;\;\;\;\;\;\;\;\;\;\;\;\;0\;\;\;\;\;\;\;\;\;\;\;\;\;\;\;\;\;\;\;\;\;\;\;0\;\;\;\;\;\;\;\;\;\;\;\;\;\;\;\;\;\;\;1 \end{array}\right]$$

#### 4.2.3 7-DOF tandem robotic arm.

Table 8 presents the DH parameter list for the 7-DOF robotic arm. The working domain of the robotic arm is analyzed using the Monte Carlo method, as shown in Fig 11(c). Based on the analysis, the objective point is set to (−0.30, 0.12, −0.11).

**Table 8:** DH parameter list of 7-DOF robotic arm.

No. Joint	*a (rad)*	*b (m)*	*c (m)*	*d (rad)*	Joint angle boundary (rad)	Target point
1	0	0.5	0	-π/2	[-3.1416, 3.1416]	(−0.30, 0.12, −0.11)
2	0	0	0.2	π/2	[-1.5708, 0.5236]	
3	0	0	0.25	-π/2	[-1.5708, 2.0944]	
4	0	0	0.3	π/2	[-1.5708, 1.5708]	
5	0	0	0.2	-π/2	[-1.5708, 1.5708]	
6	0	0	0.2	0	[-1.5708, 1.5708]	
7	0	0.05	0.1	0	[-0.5236,1.5708]	

**Fig 18:**
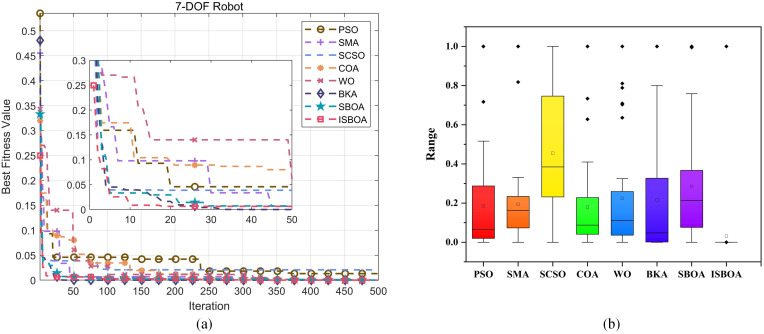
Inverse solution of convergence curve and fitness of 7-DOF robotic arm.

Fig 18(a) displays the convergence curves of the eight optimization algorithms for solving the inverse kinematics problem of the 7-DOF robotic arm. From the figure, it is clear that ISBOA achieves the best performance in terms of convergence speed, with its fitness value rapidly dropping to 6.4 × 10^−3^ within 20 iterations, significantly outperforming the other algorithms. While BKA and SBOA converge slightly slower than ISBOA, they still exhibit relatively fast convergence, stabilizing after 30 iterations. In contrast, PSO, SMA, and WO show moderate convergence speeds, stabilizing after approximately 50 iterations, but with higher final fitness values. SCSO and COA, on the other hand, have the slowest convergence speeds and fail to stabilize even after 50 iterations. In terms of convergence accuracy, ISBOA achieves the lowest final fitness value of 2.64 × 10^−7^, demonstrating the highest solution accuracy. Although the accuracies of BKA and SBOA are slightly inferior to ISBOA, they are still superior to the other algorithms. In contrast, SCSO and COA yield the highest final fitness values and the poorest accuracy. Furthermore, the convergence curves of ISBOA, BKA, and SBOA are smooth and stable. PSO and SMA exhibit slight fluctuations but remain generally stable, while the curves of SCSO and COA show pronounced fluctuations, making their results more susceptible to random factors.

The normalized boxplot in Fig 18(b) highlights ISBOA’s exceptional performance in terms of stability. The algorithm has the smallest box, a highly concentrated distribution, and almost no outliers, indicating minimal solution fluctuation and high stability across numerous experiments. SMA and COA show the second-best stability, with smaller boxes and a few outliers, maintaining relatively well-distributed fitness values. PSO and WO have slightly larger boxes with a wider range and more outlier points, reflecting average stability and higher susceptibility to random factors. Meanwhile, SCSO, BKA, and SBOA have the largest boxes with the widest distribution ranges, indicating significant fitness value fluctuations and the worst stability among the algorithms. In terms of fitness value (precision), ISBOA exhibits the lowest median, data closest to zero, and the highest precision. BKA and PSO follow closely, with medians near ISBOA and relatively high precision. SMA, COA, and WO show moderate medians and average precision. SCSO and SBOA, however, have the highest medians and the poorest fitness values, with solution quality far inferior to the other algorithms. In conclusion, ISBOA performs optimally in terms of both stability and accuracy, making it the most effective algorithm for solving the inverse kinematics problem of the 7-DOF robotic arm.

**Fig 19:**
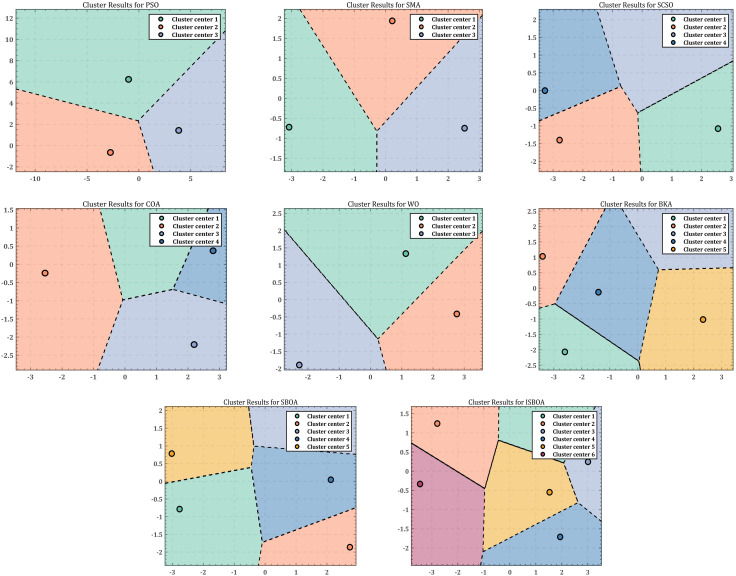
Clustering results of inverse solution of 7-DOF robotic arm.

The inverse solution results of the eight optimization algorithms are recorded 30 times, and the data are reduced using the Principal Component Analysis (PCA) method, followed by clustering with the K-means method to analyze the inverse solution diversity and performance of ISBOA. The dimensionality reduction and clustering results are displayed in Fig 19. When comparing the eight algorithms (PSO, SMA, SCSO, COA, WO, BKA, SBOA, and ISBOA), PSO and SMA identify three clustering regions. However, the clusters produced by PSO are farther apart and more uniformly distributed, whereas SMA exhibits uneven distribution, suggesting a bias in its results. WO also identifies three regions, but its solution distribution is clearly uneven, reflecting limited diversity performance. The inverse solution results of SCSO and COA are similar, with both algorithms finding four evenly distributed solution regions, offering better exploration ability but with limited coverage of the solution space. In contrast, BKA and SBOA demonstrate higher inverse solution diversity, each identifying five evenly distributed regions with strong exploration ability. However, SBOA shows some imbalance in its solution distribution. ISBOA, on the other hand, achieves the best performance by finding the most clustered regions (six) with complex boundaries. This indicates that ISBOA possesses strong exploration capabilities, fully utilizes the solution space, maintains balanced solution distribution, and achieves the widest coverage. These features make ISBOA particularly suitable for solving inverse kinematics problems involving high-dimensional and highly redundant robotic arms.

**Fig 20:**
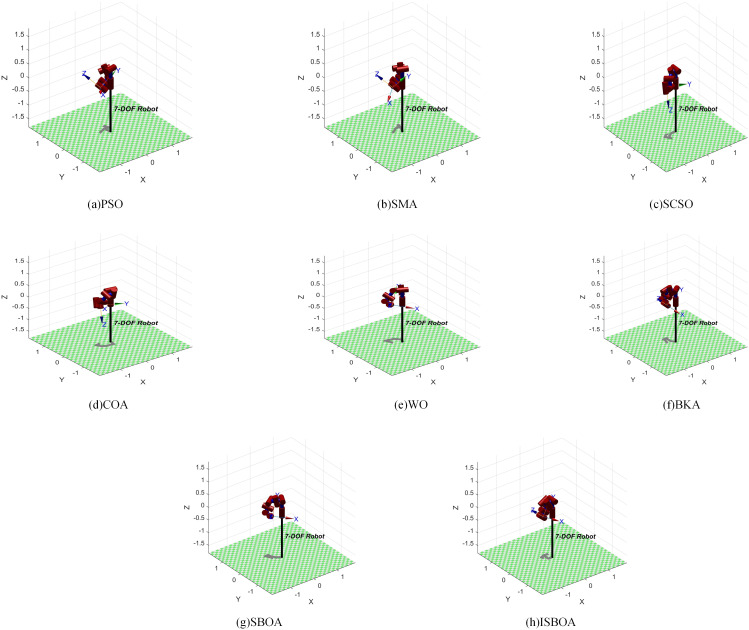
Visualization results of inverse solution of 7-DOF robotic arm.

The results of ISBOA and the other seven algorithms for solving the inverse kinematics of the 7-DOF robotic arm are shown in Table 9, where the last column lists the fitness values (with bold text indicating the optimal value). As can be seen from Table 9, the ISBOA algorithm obtains the smallest pose error of 1.2879 × 10^-13^. ISBOA consumes more computational resources compared to the PSO algorithm (4.8556s), which has the shortest running time. However, the order of magnitude difference between the two errors is 8, which is extremely critical for precision assembly tasks. These results are visualized using the Robotics Toolbox in MATLAB, as presented in Fig 20.

**Table 9:** 7-DOF robotic arm joint angle.

Optimization algorithm	Joint angle(rad)							Error	Running time
PSO	1.83	0.51	−0.68	1.57	1.08	0.57	0.77	7.0320E-05	**4.8556**
SMA	1.21	0.39	0.00	1.57	0.77	0.79	0.00	4.7969E-04	5.0854
SCSO	−3.14	0.37	0.60	1.04	−1.57	1.43	−0.37	1.7293E-02	7.2834
COA	−1.74	0.29	−1.43	1.00	−0.71	1.50	−0.52	1.4353E-04	7.3767
WO	1.10	0.51	1.18	0.88	0.04	1.50	0.51	2.1128E-06	9.2595
BKA	2.23	0.31	−0.58	1.26	0.95	1.09	0.39	3.0970E-11	8.3332
SBOA	2.65	0.23	−0.56	1.08	0.66	1.39	0.50	8.6361E-07	6.3500
ISBOA	−3.14	0.52	−1.57	1.51	1.25	0.28	1.54	**1.2879E-13**	6.3179

By substituting the inverse solution joint angles of ISBOA from Table 9 into its homogeneous transformation matrix, the resulting position matrix confirms that the joint angles computed by ISBOA accurately reach the target point.


$$A_{0}^{7}=\left[\begin{array}{c} \;\;\;0.1886\;\;\;\;\;\;0.6343\;\;\;\;\;-0\mathrm{.7497}\;\;\;\;\;\;\;-0.3\\ -0.9728\;\;\;\;\;\;0.2253\;\;\;\;\;-0.0542\;\;\;\;\;\;\;\;0.12\\ \;\;\;0.1345\;\;\;\;\;\;0.7395\;\;\;\;\;\;\;\;\;0\mathrm{.6595}\;\;\;\;\;-0.11\\ \;\;\;\;\;\;\;\;\;0\;\;\;\;\;\;\;\;\;\;\;\;\;\;\;\;\;\;0\;\;\;\;\;\;\;\;\;\;\;\;\;\;\;\;\;\;\;\;\;0\;\;\;\;\;\;\;\;\;\;\;\;\;\;\;\;\;\;1 \end{array}\right]$$

For comparison, ISBOA and the seven other algorithms (PSO, SMA, SCSO, COA, WO, BKA, and SBOA) are applied to solve the inverse kinematics problems of robotic arms with 4, 6, and 7-DOF for a single target point. Qualitative and quantitative analyses are conducted based on convergence curves and fitness errors, illustrating the superiority of ISBOA in terms of convergence speed and computational accuracy. The inverse kinematics joint angles are further analyzed using dimensionality reduction and clustering to verify the inverse solution diversity of ISBOA. Overall, the results of the simulation experiments highlight the effectiveness and applicability of ISBOA in solving inverse kinematics problems for multi-DOF robotic arms.

### 4.3 Physical validation

The simulation experiments for the inverse kinematics solutions of 4, 6, and 7-DOF robotic arms in Section 4.2 illustrate the feasibility of the ISBOA-based inverse kinematics model proposed in this paper. In the assembly process of pyrotechnic products, due to the small aperture of the outer shell and the high precision required for assembly, the robotic arm must accurately and quickly reach the target point, placing high demands on the real-time kinematics solution. While the limitations of traditional methods are discussed in the Section 1, the traditional analytical method remains the primary approach for calculating inverse solutions of robotic arms. To validate the effectiveness of the ISBOA-based inverse kinematics model in the assembly of pyrotechnic grain, this subsection compares the ISBOA model against the traditional analytical method and Newton iterative method in a physical verification setup.

**Fig 21:**
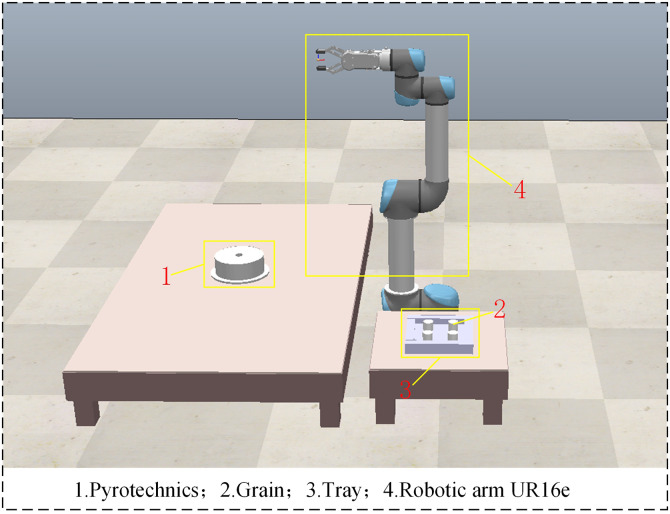
Virtual scenarios based on CoppeliaSim.

The experimental platform is constructed using MATLAB-CoppeliaSim-UR16e, where the virtual experiment is first conducted in MATLAB-CoppeliaSim. Through the identification of the pyrotechnic grain, the grasping paths are planned using the artificial potential field method, and joint angle calculations are performed using the traditional analytical method, Newton iterative method and ISBOA. Once feasibility is verified in the virtual environment, the grasping commands are sent to the physical robotic arm (UR16e) for real-world experiments. The virtual scene constructed in CoppeliaSim is shown in Fig 21.

**Fig 22:**
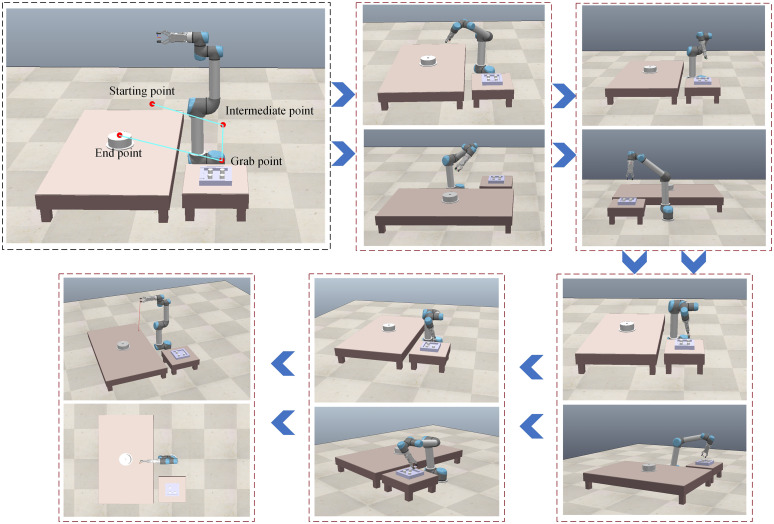
Simulation experiment based on CoppeliaSim.

After planning the grasping path using the artificial potential field method, the grasping path is obtained, as illustrated in Fig 22. Four key points of the path are identified sequentially: the starting point (−0.45, −0.05, 0.5), the middle point (0.04, −0.44, 0.5), the grasping point (0.04, −0.44, 0.3), and the ending point (−0.675, 0, 0.3). Between each pair of key points, 100 intermediate points are uniformly interpolated, resulting in a total of 300 path points. The inverse kinematics of these points are solved using both the traditional analytical method, Newton iterative method and ISBOA under identical experimental conditions. The experiments are conducted four times, and the joint angle errors relative to the target points are recorded. For readability, only the four key path points from the four experiments are presented, as shown in Table 10 (with the optimal values bolded).

**Table 10:** Comparison of simulation experiment.

Number of experiments	Target point	Method	Joint angle (rad)						Error (m)
1	(−0.45, −0.05, 0.5)	Traditional analytical method	−0.24	−1.06	1.24	0.42	−1.73	0.00	**3.1852E-11**
		Newton iterative method	−0.07	−1.05	1.31	0.74	−2.48	0.00	8.0422E-10
		ISBOA	−0.57	−0.18	−1.61	2.41	0.32	3.14	3.2435E-09
2	(0.04, −0.44, 0.5)	Traditional analytical method	1.26	−1.09	1.31	0.41	−1.59	0.00	9.5700E-07
		Newton iterative method	−1.29	−1.97	−1.27	2.88	2.47	0.00	7.7378E-11
		ISBOA	−1.31	3.11	2.11	2.32	−2.59	−0.91	**8.7651E-12**
3	(0.04, −0.44, 0.3)	Traditional analytical method	1.25	−0.80	1.67	0.24	−1.56	0.00	9.3619E-11
		Newton iterative method	−0.99	−2.43	−2.10	2.37	1.28	0.00	6.7871E-11
		ISBOA	−0.83	3.00	2.21	0.91	−0.67	3.14	**2.2672E-12**
4	(−0.675, 0, 0.3)	Traditional analytical method	−0.35	−0.41	0.62	0.69	−1.05	0.00	2.9366E-11
		Newton iterative method	−0.32	−0.36	0.54	0.73	−1.21	0.00	5.4255E-11
		ISBOA	−3.05	3.09	0.82	2.04	2.91	3.13	**9.0032E-12**

For each coordinate point, the inverse solution is calculated using three methods to derive the robotic arm’s joint angles. Subsequently, the forward kinematics of the robotic arm are used to compute the accuracy error between the robotic arm’s position and the original target point. As shown in Table 10, the ISBOA-based inverse kinematics model achieved three optimal accuracy results, indicating that its joint computation error is smaller and more uniformly distributed than that of the traditional analytical method and Newton iterative method. In the first experiment, ISBOA achieved a coordinate error of 3.2435 × 10^−9^, only slightly higher than the 3.1852 × 10^−11^ achieved by the traditional analytical method, with the difference being negligible. Additionally, for the same coordinate point, the joint angles computed by these methods are entirely different, demonstrating the redundancy of the robotic arm. For example, the sixth joint angle computed by the traditional analytical method is consistently 0, while ISBOA produced two distinct joint angles (3.14 and −0.9147), reflecting ISBOA’s wide searchability and diversity in the solution set space.

**Fig 23:**
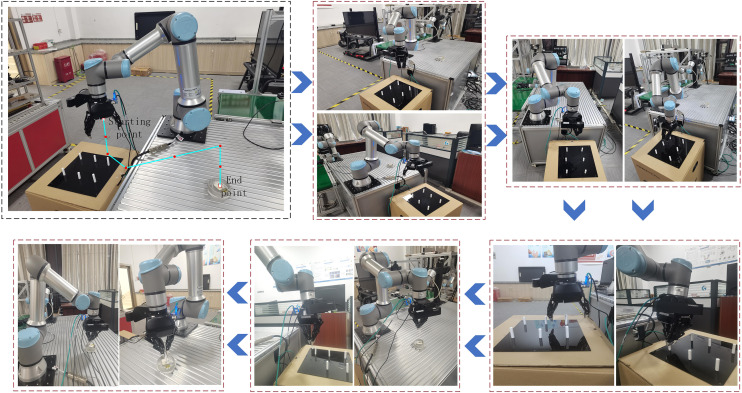
Physical validation.

After conducting the virtual simulation experiment in MATLAB-CoppeliaSim, the feasibility of the ISBOA-based inverse kinematics model for the assembly of pyrotechnic grain is demonstrated. Subsequently, its control commands are transmitted to the physical robotic arm for real-world assembly experiments. Due to strict regulations governing the use of pyrotechnic grain in the experiments, the actual grain could not be removed from their fixed location. As an alternative, 3D printing technology is used to produce substitute grain. These 3D-printed substitutes are designed to match the dimensions of the original grain, ensuring consistent geometric conditions during testing. This approach allows for the physical properties and functional use of the real grain to be approximated. The specific experimental process is illustrated in Fig 23.

In the pyrotechnic grain assembly task, the objective is to calculate the robotic arm’s joint angles and execute the task by inputting gripping path points (3D coordinate points) and solving the inverse kinematics using three methods: the traditional analytical method, Newton iterative method and ISBOA. A total of 10 and 20 experiments are conducted sequentially, and the following metrics are recorded in Table 11: the number of singular positions (instances where the robotic arm exhibits incoherent joint motion or positional issues during operation), the number of successful grips and assemblies, success rates for grips and assemblies, running time.

**Table 11:** Physical experimental data statistics.

Methods	Number of Experiments	Singular Positions	Gripping Successes	Assembly Successes	Gripping Success Rate	Assembly Success Rate	Running time (s)
Traditional analytical method	10	1	9	8	90%	80%	0.3648
	20	3	18	18	90%	90%	
Total	30	4	27	26	90%	86.7%	
Newton iterative method	10	1	9	9	90%	90%	0.0206
	20	2	19	19	95%	95%	
Total	30	3	28	28	93%	93%	
ISBOA	10	1	10	9	100%	90%	8.0851
	20	1	19	20	95%	100%	
Total	30	2	29	29	96.7%	96.7%	

When analyzing the experimental results of the traditional analytical method, Newton iterative method and ISBOA, ISBOA demonstrates significant advantages in several key indicators. Across 30 experiments, ISBOA records only 2 singular positions, compared to 4 for the traditional analytical method and 3 for Newton iterative method. This highlights ISBOA’s superior stability in inverse kinematics solving, effectively avoiding incoherent joint motions. This improvement can be attributed to ISBOA’s objective function, which optimizes the rotation of the previous target point matrix while solving the position matrix for the next target point. Furthermore, ISBOA outperforms the traditional analytical method and Newton iterative method in terms of success rates for gripping and assembly. ISBOA achieves a gripping success rate of 96.7% and an assembly success rate of 96.7%, compared to 90% and 86.7%, respectively, for the traditional analytical method. This advantage is due to ISBOA’s smooth and stable path planning, which reduces jitter of the robotic arm. Consequently, its inverse kinematics solution better satisfies the accuracy requirements of the assembly task. Under identical experimental conditions, ISBOA consistently outperforms the traditional method across all metrics, showing a clear trend of superiority. The Newton iterative method is the most computationally efficient with a running time of 0.0206s. However, ISBOA consumes more computational resources. This is due to the fact that both methods are essentially optimization iterations, but the principles employed are different. ISBOA uses stochastic optimization, and its computational accuracy cannot be ignored despite the longer running time under the objective function and constraints. For example, in Table 10, ISBOA achieves the three highest accuracies out of four points, outperforming traditional analytic and Newton iterative methods by 1–2 orders of magnitude.

Overall, the ISBOA algorithm demonstrates significant advantages over the traditional analytical method and Newton iterative method in practical applications. Based on its optimization principles, ISBOA effectively avoids singularities and joint motion discontinuities in robotic arm motion. It also exhibits stronger global and adaptive path planning capabilities for precision assembly task, ensuring the stable completion of tasks. The high success rates in gripping and assembly reflect ISBOA’s computational accuracy and adaptability to uncertainties in real-world environments. In contrast, while the traditional analytical method is computationally efficient, it is constrained by the kinematic model of the robotic arm, making it less effective in avoiding singularities. For Newton iterative method, the lower success rates for gripping and assembly may stem from non-smooth joint angle transitions or unstable solutions. Therefore, ISBOA emerges as a superior choice for complex tasks requiring high precision and high success rates, such as the assembly of pyrotechnic grain.

## 5. Summary and prospect

This paper focuses on the development of flexible automatic assembly technology for multi-species, small-batch pyrotechnic products, addressing the key challenges posed by the small diameter of shell holes and the high precision required in the assembly of pyrotechnic grain. During the assembly process, the robotic arm must accurately and swiftly reach target positions to execute high-precision tasks, which imposes strict requirements on the real-time kinematic solution. To meet these demands, this study constructs an inverse kinematics objective function for the robotic arm and reformulates the inverse solution problem as a constrained optimization problem. To address the limitations of the traditional SBOA algorithm, such as insufficient exploration of the solution space, slow convergence speed, and susceptibility to local optima when solving the inverse kinematics of multi-DOF robotic arms, an improved strategy is proposed. Specifically, the SBOA algorithm is enhanced by introducing an oppositional variational perturbation strategy, a golden sine development strategy, and an evolutionary strategy, culminating in the development of a novel optimization algorithm, ISBOA.

Initially, numerical experiments are conducted using the CEC2017 benchmark functions. The results indicate that ISBOA outperforms the original SBOA and other comparative algorithms in terms of convergence speed, accuracy, and the ability to escape local optima, validating the effectiveness of the proposed improvements. These findings further substantiate the optimization performance of ISBOA from a mathematical perspective. Subsequently, simulation experiments are performed on robotic arms with 4, 6, and 7-DOF based on the inverse kinematics solution model developed with ISBOA. The experimental results are analyzed qualitatively and quantitatively through convergence curves, fitness errors, and the diversity of inverse solutions, demonstrating the reliability and effectiveness of ISBOA in solving inverse kinematics for multi-DOF robotic arms. To further validate ISBOA’s practical applicability, a MATLAB-CoppeliaSim-UR16e experimental platform is constructed for the assembly of pyrotechnic grain. Simulation experiments are first conducted in MATLAB-CoppeliaSim, followed by physical validation using a UR16e robotic arm. The experimental results, which compared ISBOA with traditional analytical methods and Newton iterative methods, are analyzed in terms of assembly accuracy, singular position handling, gripping success rate, overall assembly success rate and running time. The findings highlight the significant advantages and strong engineering potential of ISBOA-based inverse kinematics models in the pyrotechnic grain assembly process.

Overall, this paper verifies the effectiveness and success of the inverse kinematics solution technology of the robotic arm based on the ISBOA algorithm. However, this article still has the following limitations, which will also be the future working direction. Firstly, the mechanism of ISBOA will be deeply analyzed from the theoretical level, better optimization strategies will be studied to improve computing performance, and more test sets will be supplemented for verification. Secondly, the experimental analysis shows that there is a trade-off between efficiency and accuracy in the method proposed in this paper, and real-time performance is its drawback. Therefore, it is feasible to realize the real-time calculation of the inverse kinematics of the robotic arm by using neural networks. Then, the physical experiments in this paper are carried out in an experimental environment. For the uncertainty of pyrotechnic pillars assembly in real environment, future work should be combined with more automation techniques, such as reinforcement learning, to conduct research in real work scenarios. Finally, given the complexity of precision assembly tasks, inverse kinematics solution techniques for human-robot collaborative robots are worth exploring in depth.
